# An integrated analysis of lymphocytic reaction, tumour molecular characteristics and patient survival in colorectal cancer

**DOI:** 10.1038/s41416-020-0780-3

**Published:** 2020-03-11

**Authors:** Koichiro Haruki, Keisuke Kosumi, Peilong Li, Kota Arima, Juha P. Väyrynen, Mai Chan Lau, Tyler S. Twombly, Tsuyoshi Hamada, Jonathan N. Glickman, Kenji Fujiyoshi, Yang Chen, Chunxia Du, Chunguang Guo, Sara A. Väyrynen, Andressa Dias Costa, Mingyang Song, Andrew T. Chan, Jeffrey A. Meyerhardt, Reiko Nishihara, Charles S. Fuchs, Li Liu, Xuehong Zhang, Kana Wu, Marios Giannakis, Jonathan A. Nowak, Shuji Ogino

**Affiliations:** 1 Program in MPE Molecular Pathological Epidemiology, Department of Pathology, Brigham and Women’s Hospital and Harvard Medical School, Boston, MA USA; 2 Department of Clinical Laboratory, The Second Hospital of Shandong University, Jinan, China; 3 Cancer and Translational Medicine Research Unit, Medical Research Center Oulu, Oulu University Hospital, and University of Oulu, Oulu, Finland; 4 Department of Pathology, Beth Israel Deaconess Medical Center, Boston, MS USA; 5 Department of Medical Oncology, Dana-Farber Cancer Institute and Harvard Medical School, Boston, MA USA; 6 Department of Nutrition, Harvard T.H. Chan School of Public Health, Boston, MA USA; 7 Clinical and Translational Epidemiology Unit, Massachusetts General Hospital and Harvard Medical School, Boston, MA USA; 8 Division of Gastroenterology, Massachusetts General Hospital, Boston, MA USA; 9 Channing Division of Network Medicine, Department of Medicine, Brigham and Women’s Hospital and Harvard Medical School, Boston, MA USA; 10 Department of Immunology and Infectious Diseases, Harvard T.H. Chan School of Public Health, Boston, MA USA; 11 Department of Epidemiology, Harvard T.H. Chan School of Public Health, Boston, MA USA; 12 Department of Biostatistics, Harvard T.H. Chan School of Public Health, Boston, MA USA; 13 Yale Cancer Center, New Haven, CT USA; 14 Department of Medicine, Yale School of Medicine, New Haven, CT USA; 15 Smilow Cancer Hospital, New Haven, CT USA; 16 Department of Epidemiology and Biostatistics, and the Ministry of Education Key Lab of Environment and Health, School of Public Health, Huazhong University of Science and Technology, Wuhan, China; 17 Broad Institute of MIT and Harvard, Cambridge, MA USA; 18 Department of Medicine, Brigham and Women’s Hospital and Harvard Medical School, Boston, MA USA; 19 Cancer Immunology and Cancer Epidemiology Programs, Dana-Farber Harvard Cancer Center, Boston, MA USA

**Keywords:** Cancer microenvironment, Cancer microenvironment, Lymphocytes, Prognostic markers

## Abstract

**Background:**

Histological lymphocytic reaction is regarded as an independent prognostic marker in colorectal cancer. Considering the lack of adequate statistical power, adjustment for selection bias and comprehensive tumour molecular data in most previous studies, we investigated the strengths of the prognostic associations of lymphocytic reaction in colorectal carcinoma by utilising an integrative database of two prospective cohort studies.

**Methods:**

We examined Crohn’s-like reaction, intratumoural periglandular reaction, peritumoural reaction and tumour-infiltrating lymphocytes in 1465 colorectal carcinoma cases. Using covariate data of 4420 colorectal cancer cases in total, inverse probability-weighted Cox proportional hazard regression model was used to control for selection bias (due to tissue availability) and potential confounders, including stage, MSI status, LINE-1 methylation, PTGS2 and CTNNB1 expression, *KRAS*, *BRAF* and *PIK3CA* mutations, and tumour neoantigen load.

**Results:**

Higher levels of each lymphocytic reaction component were associated with better colorectal cancer-specific survival (*P*_trend_ < 0.002). Compared with cases with negative/low intratumoural periglandular reaction, multivariable-adjusted HRs were 0.55 (95% CI, 0.42–0.71) in cases with intermediate reaction and 0.20 (95% CI, 0.12–0.35) in cases with high reaction. These relationships were consistent in strata of MSI status or neoantigen loads (*P*_interaction_ > 0.2).

**Conclusions:**

The four lymphocytic reaction components are prognostic biomarkers in colorectal carcinoma.

## Background

Host immune response in the tumour microenvironment plays a critical role in regulating cancer initiation and progression.^1–4^ Histological lymphocytic reaction that reflects host immune response to tumour cells can be evaluated by Crohn’s-like lymphoid reaction, peritumoural lymphocytic reaction, intratumoural periglandular reaction and tumour-infiltrating lymphocytes (TIL).^5^ Accumulating evidence indicates that colorectal cancer with microsatellite instability (MSI)-high status is characterised by higher lymphocytic reaction, because of potentially immunogenic neoantigens generated by frameshift mutations due to defective DNA mismatch repair.^5–8^ In fact, higher neoantigen load has been positively associated with overall lymphocytic infiltration, TIL, memory T cells and better colorectal cancer-specific survival.^9,10^ In addition, the specific tumour molecular alterations, including PTGS2 expression,^11^ nuclear CTNNB1 expression,^12^ CpG island methylator phenotype (CIMP) status^13^ and long-interspersed nucleotide element-1 (LINE-1) methylation levels,^14^ can modify the antitumour immune response, and all these factors have been associated with colorectal cancer mortality.^15–18^ However, none of the studies has taken these molecular features into account in the prognostic analysis of antitumour immune response. Therefore, a comprehensive study focusing on the prognostic role of lymphocytic reaction and its relationship with the aforementioned molecular features is needed.

In this study, we utilised two large US-nationwide prospective cohort studies with covariate data of 4420 colorectal cancer cases, and a molecular pathological epidemiology database of 1465 cases, to evaluate the relationships between lymphocytic reaction patterns and patient survival. We hypothesised that more intense host lymphocytic reaction to colorectal cancer might be associated with a favourable clinical outcome, after adjusting for other potential confounders including neoantigen load. To reduce potential bias due to the availability of tumour tissue, we utilised inverse probability-weighting (IPW) method^19–22^ (on the 4420 cases), which has not been used in the previous prognostic studies of immune response to tumour. In addition, we examined statistical interactions between lymphocytic reaction and MSI status or neoantigen load.

## Materials and methods

### Study population

We collected data on colorectal cancer cases within two prospective cohort studies in the United States, the Nurses’ Health Study (NHS, 121,701 women aged 30–55 years followed since 1976) and the Health Professionals Follow-up Study (HPFS, 51,529 men aged 40–75 years followed since 1986).^23^ Every 2 years, study participants have been sent follow-up questionnaires to collect information on lifestyle factors and medical history of physician-confirmed diseases including colorectal cancer. The National Death Index was used to ascertain deaths of study participants and identify unreported lethal colorectal cancer cases. Participating physicians reviewed medical records to confirm diagnosis of colorectal cancer, and to record tumour characteristics (e.g. size, location and the American Joint Committee on Cancer tumour, node and metastases (TNM) classification), and causes of deaths for participants who were deceased. Formalin-fixed paraffin-embedded (FFPE) tissue blocks were collected from hospitals where participants diagnosed with colorectal cancer had undergone tumour resection. We included 1465 patients with available data on at least one of four histopathological lymphocytic reactions. We included both colon and rectal carcinomas based on the colorectal continuum model.^24^ Patients were followed until death or the end of follow-up (January 1, 2014 for HPFS; May 31 for NHS), whichever came first. Informed consent was obtained from all study participants. This study was approved by the institutional review boards at Harvard T.H. Chan School of Public Health and Brigham and Women’s Hospital (Boston, MA), and those of participating registries as required.

### Histopathological evaluations

**Fig. 1:**
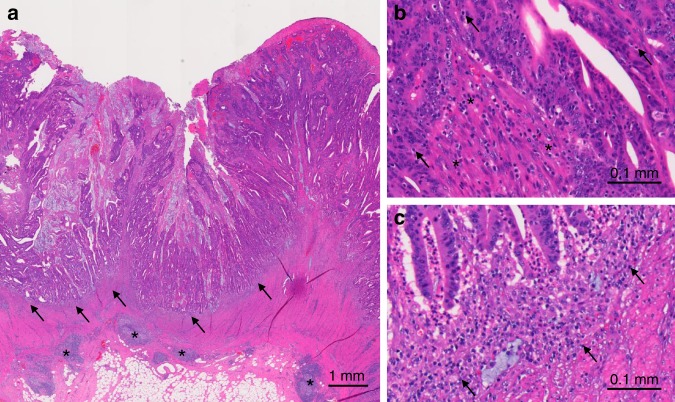
The four components of lymphocytic reaction against colorectal cancer. **a** Peritumoural lymphocytic reaction (arrows) and Crohn-like lymphoid reaction (asterisks). **b** Tumour-infiltrating lymphocytes (arrows) and intratumoural periglandular reaction (asterisks). **c** Peritumoural lymphocytic reaction (arrows).

FFPE blocks of tumour tissues were collected from hospitals throughout the United States, where colorectal cancer patients had undergone surgical resection. A single pathologist (S.O.), who was unaware of other data, reviewed haematoxylin- and eosin-stained tissue sections, and recorded histopathological findings, including tumour differentiation and lymphocytic reaction components, as previously described.^5^ Tumour differentiation was categorised as well to moderate vs. poor (>50% vs. ≤50% gland formation, respectively). Four components of lymphocytic reactions (Crohn’s-like lymphoid reaction, peritumoural lymphocytic reaction, intratumoural periglandular reaction and TIL) were examined (Fig. 1). Crohn’s-like lymphoid reaction was defined as transmural lymphoid reaction. Peritumoural lymphocytic reaction was defined as discrete lymphoid reaction surrounding a tumour mass. Intratumoural periglandular reaction was defined as lymphocytic reaction in tumour stroma within a tumour mass. TIL was defined as lymphocytes on top of cancer cells. For any given tumour, each of the four lymphocytic reaction components was scored as 0, 1+, 2+ and 3+, and graded as negative/low (0), intermediate (1+) and high (2+ and 3+) as previously described.^5,25^ A review of 398 randomly selected cases between two independent pathologists (S.O. and J.N.G.) showed good concordance on grading of histopathological features, including lymphocytic reaction to tumour.^5^ For the analyses of lymphocytic reaction and patient survival in strata of tumour neoantigen load, each of the four lymphocytic reaction components was graded as negative/low (0) and intermediate/high (1+, 2+ and 3+). The overall lymphocytic reaction score (0–12) was calculated as the sum of scores for the above four reaction components, and was graded as low (0–2), intermediate (3–6) and high (7–12).

### Analyses of microsatellite instability (MSI), DNA methylation, *KRAS*, *BRAF* and *PIK3CA* mutations and neoantigen load

Genomic DNA was extracted from colorectal cancer tissue in whole-tissue sections of archival FFPE tissue blocks. MSI status was evaluated using ten microsatellite markers (D2S123, D5S346, D17S250, BAT25, BAT26, BAT40, D18S55, D18S56, D18S67 and D18S487), as previously described.^24^ MSI-high status was defined as the presence of instability in ≥30% of the markers, and non-MSI-high as instability in <30% of the markers, as previously described.^26^ DNA methylation was measured in eight CIMP-specific promoters (*CACNA1G*, *CDKN2A*, *CRABP1*, *IGF2*, *MLH1*, *NEUROG1*, *RUNX3* and *SOCS1*) and in LINE-1.^27,28^ CIMP-high was defined as ≥6 methylated promoters of eight promoters, and CIMP-low/negative as <6 methylated promoters. PCR and pyrosequencing were performed for *KRAS* (codons 12, 13, 61 and 146), *BRAF* (codon 600) and *PIK3CA* (exons 9 and 20), as previously described.^24^ Neoantigen load, the number of proteins that likely give rise to immunogenic peptides in the tumour microenvironment, was predicted for 505 cases, by using a neoantigen prediction pipeline for somatic mutations based on whole-exome sequencing, and identifying peptides that bind to personal human leukocyte antigen (HLA) molecules with high affinity (<500 nM), as previously described.^29^ Using NetMHCpan (version 2.4, Technical University of Denmark, DK-2800 Lyngby, Denmark),^30^ we predicted the binding affinities of all possible 9- and 10-mer mutant peptides to the corresponding HLA alleles inferred by the POLYSOLVER algorithm.

### Immunohistochemistry for PTGS2 (cyclooxygenase-2), CTNNB1 (beta-catenin) and CD274 (PDCD1 ligand 1)

We constructed tissue microarrays of colorectal cancer cases with sufficient tissue materials, including up to four tumour cores from each case.^31^ Immunohistochemical analyses for PTGS2 (cyclooxygenase-2), nuclear CTNNB1 (beta-catenin) and CD274 (programmed death-ligand 1, PDCD1 ligand 1, PD-L1) were performed using an anti-PTGS2 antibody (dilution, 1:300, Cayman Chemical, Ann Arbor, MI), an anti-CTNNB1 antibody (dilution, 1:400, BD Transduction Laboratories, Franklin Lakes, NJ) and an anti-CD274 antibody (dilution, 1:50, eBioscience, San Diego, CA), respectively, as previously described.^17,31,32^

### Statistical analysis

All statistical analyses were performed using SAS software (version 9.4, SAS Institute, Cary, NC), and all *P* values were two-sided. We used a two-sided α level of 0.005 for our primary hypothesis testing.^33^ Our primary hypothesis testing was assessment of associations of four lymphocytic reaction components (negative/low vs. intermediate vs. high) with colorectal cancer-specific survival in the Cox proportional hazard regression model. All other analyses, including evaluation of individual hazard ratio (HR) estimates, assessment of stratum-specific risk estimates and of interaction with MSI status and neoantigen load, represented secondary analyses.

To assess the association between ordinal categories of the level of lymphocytic reaction (negative/low, intermediate and high) and other categorical variables, the chi-square test was performed. To compare continuous variables (age and LINE-1), an analysis of variance assuming equal variances was performed.

We utilised inverse probability-weighting (IPW) method using covariate data of 4420 colorectal cancer cases with or without tumour tissue, to adjust for selection bias due to tissue availability.^19^ Multivariable IPW-adjusted Cox proportional hazard regression models were used to adjust for potential confounders. The multivariable IPW-adjusted Cox proportional hazard regression models initially included sex (female vs. male), age at diagnosis (continuous), year of diagnosis (continuous), family history of colorectal cancer in any first-degree relative (present vs. absent), tumour location (proximal colon vs. distal colon vs. rectum), disease stage (I vs. II vs. III vs. IV), tumour differentiation (well/moderate vs. poor), MSI status (MSI-high vs. non-MSI-high), CIMP (low/negative vs. high), *KRAS* (mutant vs. wild type), *BRAF* (mutant vs. wild type), *PIK3CA* (mutant vs. wild type), LINE-1 methylation level (continuous), PTGS2 expression (positive vs. negative) and nuclear CTNNB1 expression (positive vs. negative). A backward elimination was conducted with a threshold *P* of 0.05 to select variables for the final models. Cases with missing data (family history of colorectal cancer in a first-degree relative (0.3%) and tumour location (0.4%)) were included in the majority category of a given categorical covariate to limit the degrees of freedom of the models. For the cases with missing data on LINE-1 methylation (13.0%), we assigned a separate indicator variable. For cases with missing information on MSI status (11.6%), CIMP status (14.6%), *KRAS* mutation (14.6%), *BRAF* mutation (10.7%), *PIK3CA* mutation (17.1%), PTGS2 (15.7%) and CTNNB1 (35.8%), we assigned a separate missing indicator variable. We confirmed that excluding the cases with missing information in any of the covariates did not substantially alter the results (data not shown). For the analyses using a subset of cases with available neoantigen load data, we included neoantigen load (continuous) to the multivariable IPW-adjusted Cox proportional hazard regression models in addition to the aforementioned potential confounders. The proportionality of hazards assumption in colorectal cancer survival was assessed by a time-varying covariate, which was an interaction term of survival time and the level of lymphocytic reaction (*P* > 0.27). We observed evidence on violation of this assumption in the hazards for four lymphocytic reaction components and the overall lymphocytic score in overall survival. However, the Schoenfeld residual plots supported the proportionality of hazards during most of the follow-up period up to 10 years (data not shown), and thus, we used Cox regression models limiting the follow-up period to 10 years. Cumulative survival probabilities were estimated using the IPW-adjusted Kaplan–Meier method, and a linear trend in survival probabilities across ordinal categories of the level of lymphocytic reaction was assessed using the weighted log-rank test for trend. For analyses of colorectal cancer-specific survival, participants were censored at the time of deaths from other causes.

In secondary analyses, we assessed the statistical interaction between levels of four lymphocytic reaction components (negative/low vs. intermediate vs. high) and each of following features: MSI status (high vs. non-high), neoantigen load (high vs. low), year of diagnosis (1995 or before vs. 1996–2000 vs. 2001–2008) and tumour location (proximal colon vs. distal colon vs. rectum), using the Wald test in the multivariable-adjusted Cox proportional hazard regression model for colorectal cancer mortality. We estimated HR for a unit increase of each lymphocytic reaction component in strata of MSI status, neoantigen load, year of diagnosis and tumour location using re-parameterisation of the interaction term in a single regression model.^27^

In all survival Cox regression analyses, the IPW method was applied to reduce the potential bias due to the availability of tumour tissue.^19–21^ Using the multivariable logistic regression model for the entire dataset of colorectal cancer cases (regardless of available tissue), we estimated the probability of the availability of tumour tissue, as previously described.^25^ Each patient with complete data was weighted by the inverse probability. Weights greater than the 95th percentile were truncated and set to the value of the 95th percentile to reduce outlier effects.^21^ We confirmed that the results without weight truncation did not change substantially (data not shown). The Cox regression analyses without IPW yielded similar results to the IPW-adjusted model.

## Results

We used covariate data of 4420 rectal and colon carcinoma cases in the two prospective cohort studies for the inverse probability- weighting (IPW) method to adjust for selection bias due to tissue availability.^19^ In 1465 cases, we examined lymphocytic reaction patterns: tumour-infiltrating lymphocytes (TIL, 1461 cases), intratumoural periglandular reaction (1462 cases), peritumoural lymphocytic reaction (1456 cases) and Crohn’s-like lymphoid reaction (1195 cases) (Table 1; Supplementary Table S1). All of the four lymphocytic reaction components were positively associated with proximal location, early disease stage, well-to-moderate tumour differentiation, MSI-high status, CIMP-high status, LINE-1 hypermethylation, *BRAF* mutation, negative nuclear CTNNB1 expression and high neoantigen load (all *P* < 0.005). During the median follow-up time of 12.3 years (interquartile range, 8.0–16.6 years) for all censored patients, there were 885 all-cause deaths, including 432 colorectal cancer-specific deaths.

**Table 1:** Clinical, pathological and molecular characteristics of colorectal cancer cases according to the intratumoural periglandular reaction to colorectal cancer and tumour-infiltrating lymphocytes.

	Intratumoural periglandular reaction					Tumour-infiltrating lymphocytes				
Characteristic^a^	No. of cases (*N* = 1462)	Negative/low (*N* = 193)	Intermediate (*N* = 1085)	High (*N* = 184)	*P*-value^b^	No. of cases (*N* = 1461)	Negative/low (*N* = 1095)	Intermediate (*N* = 219)	High (*N* = 147)	*P*-value^b^
*Sex*					0.51					0.0039
Female (NHS)	820 (56%)	114 (59%)	599 (55%)	107 (58%)		821 (56%)	588 (54%)	139 (63%)	94 (64%)	
Male (HPFS)	642 (44%)	79 (41%)	486 (45%)	77 (42%)		640 (44%)	507 (46%)	80 (37%)	53 (36%)	
Mean age ± SD (years)	69.0 ± 9.0	70.3 ± 9.2	68.4 ± 9.0	71.1 ± 8.1	<0.0001	69.0 ± 9.0	68.5 ± 9.0	70.5 ± 8.8	70.3 ± 8.4	0.0017
*Year of diagnosis*					<0.0001					0.0076
1995 or before	520 (36%)	32 (17%)	445 (41%)	43 (23%)		520 (36%)	411 (38%)	60 (27%)	49 (33%)	
1996–2000	440 (30%)	44 (23%)	337 (31%)	59 (32%)		439 (30%)	324 (30%)	63 (29%)	52 (35%)	
2001–2008	502 (34%)	117 (61%)	303 (28%)	82 (45%)		502 (34%)	360 (33%)	96 (44%)	46 (31%)	
*Family history of colorectal cancer in first-degree relative(s)*					0.44					0.26
Absent	1174 (81%)	160 (83%)	871 (81%)	143 (78%)		1172 (80%)	889 (81%)	169 (78%)	114 (78%)	
Present	283 (19%)	33 (17%)	209 (19%)	41 (22%)		284 (20%)	202 (19%)	49 (22%)	33 (22%)	
*Tumour location*					<0.0001					<0.0001
Caecum	256 (18%)	21 (11%)	197 (18%)	38 (21%)		256 (18%)	171 (16%)	51 (23%)	34 (23%)	
Ascending to transverse colon	447 (31%)	67 (35%)	298 (28%)	82 (45%)		446 (31%)	280 (26%)	85 (39%)	81 (55%)	
Descending to sigmoid colon	439 (30%)	62 (32%)	333 (31%)	44 (24%)		440 (30%)	366 (34%)	48 (22%)	26 (18%)	
Rectum	314 (22%)	41 (21%)	253 (23%)	20 (11%)		313 (22%)	273 (25%)	34 (16%)	6 (4.1%)	
*AJCC disease stage*					<0.0001					<0.0001
I	343 (26%)	29 (17%)	258 (26%)	56 (33%)		343 (26%)	248 (25%)	58 (29%)	37 (26%)	
II	427 (32%)	46 (26%)	310 (32%)	71 (42%)		427 (32%)	289 (29%)	65 (33%)	73 (52%)	
III	371 (28%)	53 (30%)	282 (29%)	36 (21%)		370 (28%)	290 (29%)	56 (28%)	24 (17%)	
IV	184 (14%)	46 (26%)	131 (13%)	7 (4.1%)		185 (14%)	158 (16%)	21 (11%)	6 (4.3%)	
*Tumour differentiation*					<0.0001					<0.0001
Well to moderate	1,299 (90%)	173 (90%)	992 (92%)	134 (73%)		1,298 (90%)	1,017 (94%)	183 (84%)	98 (67%)	
Poor	151 (10%)	19 (9.9%)	82 (7.6%)	50 (27%)		151 (10%)	66 (6.1%)	36 (16%)	49 (33%)	
*MSI status*					<0.0001					<0.0001
Non-MSI-high	881 (83%)	137 (92%)	669 (87%)	75 (54%)		881 (83%)	729 (93%)	111 (66%)	41 (38%)	
MSI-high	176 (17%)	12 (8.1%)	100 (13%)	64 (46%)		176 (17%)	52 (6.7%)	58 (34%)	66 (62%)	
*CIMP status*					<0.0001					<0.0001
Low/negative	832 (82%)	129 (89%)	635 (86%)	68 (53%)		832 (82%)	695 (92%)	96 (62%)	41 (41%)	
High	183 (18%)	16 (11%)	106 (14%)	61 (47%)		183 (18%)	63 (8.3%)	60 (38%)	60 (59%)	
Mean LINE-1 methylation level ± SD (%)	63.2 ± 9.9	63.9 ± 10.2	62.6 ± 9.7	66.4 ± 10.6	<0.0001	63.2 ± 9.9	62.5 ± 9.8	64.8 ± 9.4	65.7 ± 10.8	0.0001
*KRAS* mutation					0.12					0.028
Wild type	586 (58%)	72 (52%)	435 (58%)	79 (65%)		586 (58%)	421 (56%)	94 (61%)	71 (69%)	
Mutant	423 (42%)	66 (48%)	314 (42%)	43 (35%)		423 (42%)	332 (44%)	59 (39%)	32 (31%)	
*BRAF* mutation					<0.0001					<0.0001
Wild type	893 (84%)	128 (86%)	672 (87%)	93 (68%)		893 (84%)	709 (90%)	122 (73%)	62 (58%)	
Mutant	167 (16%)	20 (14%)	103 (13%)	44 (32%)		167 (16%)	77 (9.8%)	45 (27%)	45 (42%)	
*PIK3CA* mutation					0.38					0.82
Wild type	829 (84%)	118 (82%)	609 (85%)	102 (80%)		829 (84%)	614 (83%)	133 (85%)	82 (85%)	
Mutant	161 (16%)	26 (18%)	110 (15%)	25 (20%)		161 (16%)	123 (17%)	24 (15%)	14 (15%)	
*CD274 (PD-L1) expression score*					0.092					0.039
0	69 (10%)	7 (7.3%)	51 (10%)	11 (14%)		69 (10%)	39 (7.8%)	18 (16%)	12 (17%)	
1	195 (29%)	30 (31%)	139 (28%)	26 (32%)		195 (29%)	140 (28%)	37 (33%)	18 (26%)	
2	192 (28%)	36 (38%)	131 (26%)	25 (31%)		192 (28%)	142 (28%)	28 (25%)	22 (31%)	
3	192 (28%)	18 (19%)	158 (31%)	16 (20%)		192 (28%)	153 (31%)	24 (22%)	15 (21%)	
4	33 (4.9%)	5 (5.2%)	25 (5.0%)	3 (3.7%)		33 (4.9%)	26 (5.2%)	4 (3.6%)	3 (4.3%)	
*PTGS2 (cyclooxygenase-2) expression*					0.028					0.016
Negative	377 (38%)	59 (44%)	266 (36%)	52 (47%)		377 (38%)	264 (36%)	64 (43%)	49 (49%)	
Positive	613 (62%)	76 (56%)	478 (64%)	59 (53%)		613 (62%)	477 (64%)	84 (57%)	52 (51%)	
*Nuclear CTNNB1 (beta-catenin) expression*					<0.0001					<0.0001
Negative	938 (64%)	124 (64%)	669 (62%)	145 (79%)		938 (64%)	663 (61%)	165 (75%)	110(75%)	
Positive	524 (36%)	69 (36%)	416 (38%)	39 (21%)		523 (36%)	432 (39%)	54 (25%)	37 (25%)	
*Neoantigen load*					<0.0001					<0.0001
Q1 (lowest)	125 (25%)	29 (34%)	84 (24%)	12 (16%)		125 (25%)	104 (29%)	20 (22%)	1 (1.8%)	
Q2	123 (24%)	19 (22%)	92 (27%)	12 (16%)		123 (24%)	104 (29%)	14 (15%)	5 (8.8%)	
Q3	129 (26%)	29 (34%)	86 (25%)	14 (19%)		129 (26%)	100 (28%)	19 (20%)	10 (18%)	
Q4 (highest)	128 (25%)	9 (10%)	82 (24%)	37 (49%)		128 (25%)	47 (13%)	40 (43%)	41 (72%)	

*AJCC* American Joint Committee on Cancer, *CIMP* CpG island methylator phenotype, *HPFS* health professionals follow-up study, *LINE-1* long-interspersed nucleotide element-1, *MSI* microsatellite instability, *NHS* nurses’ health study, *SD* standard deviation

^a^Percentage indicates the proportion of patients with a specific clinical, pathological or molecular characteristic among all patients or in strata of lymphocytic reaction to colorectal cancer.

^b^To assess associations between the categories (negative/low, intermediate and high) of intratumoural periglandular reaction to colorectal cancer or tumour-infiltrating lymphocytes, and categorical data, the chi-square test was performed. To compare age, and LINE-1 methylation level, an analysis of variance was performed.

**Fig. 2:**
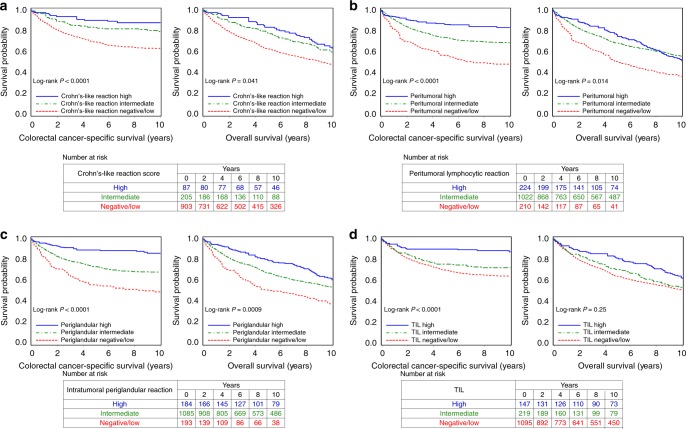
Inverse probability weighting-adjusted Kaplan–Meier survival analyses of colorectal cancer patients according to lymphocytic reaction components. The *P*-values were calculated using the weighted log-rank test for trend (two-sided). Crohn’s-like lymphoid reaction (**a**), peritumoural lymphocytic reaction (**b**), intratumoural periglandular reaction (**c**) and tumour-infiltrating lymphocytes (TIL) (**d**).

To test our primary hypothesis, we examined the relationship between each lymphocytic reaction component and patient mortality (Table 2). Higher levels of each component were associated with better cancer-specific survival (*P*_trend_ < 0.002) and better overall survival (*P*_trend_ < 0.009) in multivariable Cox regression analyses. Compared with cases with negative/low intratumoural periglandular reaction, multivariable-adjusted HRs for colorectal cancer-specific mortality were 0.55 (95% confidence interval (CI), 0.42–0.71) in cases with intermediate reaction, and 0.20 (95% CI, 0.12–0.35) in cases with high reaction. The Cox regression analyses without IPW yielded similar results to the IPW-adjusted model (Supplementary Table S2). When we adjusted for neoantigen load, as well as MSI, these findings remained largely unchanged (*P*_trend_ < 0.1 for cancer-specific survival and *P*_trend_ < 0.02 for overall survival, Supplementary Table S3). In Kaplan–Meier survival analyses, each lymphocytic reaction component was positively associated with favourable colorectal cancer-specific survival (*P* < 0.0001 by the log-rank test for trend, Fig. 2).

**Table 2:** Lymphocytic reaction components and patient survival.

		Colorectal cancer-specific survival			Overall survival		
	No. of cases	No. of events	Univariable HR (95% CI)^a^	Multivariable HR (95% CI)^a,b^	No. of events	Univariable HR (95% CI)^a^	Multivariable HR (95% CI)^a,b^
*Crohn’s-like lymphoid reaction*							
Negative/low	903	305	1 (referent)	1 (referent)	563	1 (referent)	1 (referent)
Intermediate	205	37	0.48 (0.34–0.70)	0.59 (0.40–0.85)	108	0.66 (0.51–0.85)	0.72 (0.55–0.93)
High	87	10	0.28 (0.14–0.54)	0.27 (0.12–0.58)	54	0.56 (0.38–0.81)	0.51 (0.33–0.78)
*P*_trend_^c^			<0.0001	0.0005		<0.0001	0.0001
*Peritumoural lymphocytic reaction*							
Negative/low	210	107	1 (referent)	1 (referent)	146	1 (referent)	1 (referent)
Intermediate	1022	290	0.52 (0.41–0.66)	0.54 (0.42–0.70)	611	0.56 (0.45–0.70)	0.62 (0.49–0.77)
High	224	32	0.25 (0.16–0.38)	0.28 (0.18–0.45)	124	0.55 (0.41–0.72)	0.55 (0.40–0.74)
*P*_trend_^c^			<0.0001	<0.0001		<0.0001	<0.0001
*Intratumoural perigrandular reaction*							
Negative/low	193	91	1 (referent)	1 (referent)	124	1 (referent)	1 (referent)
Intermediate	1085	315	0.55 (0.43–0.70)	0.55 (0.42–0.71)	661	0.62 (0.49–0.78)	0.68 (0.53–0.86)
High	184	24	0.20 (0.12–0.32)	0.20 (0.12–0.35)	98	0.45 (0.32–0.62)	0.43 (0.30–0.62)
*P*_trend_^c^			<0.0001	<0.0001		<0.0001	<0.0001
*Tumour-infiltrating lymphocytes*							
Negative/low	1095	356	1 (referent)	1 (referent)	670	1 (referent)	1 (referent)
Intermediate	219	57	0.77 (0.57–1.04)	0.72 (0.52–0.99)	126	0.91 (0.72–1.15)	0.81 (0.62–1.05)
High	147	17	0.30 (0.18–0.49)	0.33 (0.19–0.58)	87	0.65 (0.49–0.88)	0.60 (0.41–0.86)
*P*_trend_^c^			<0.0001	0.0014		0.0066	0.0080

*CI* confidence interval, *HR* hazard ratio, *IPW* inverse probability weighting.

^a^IPW was applied to reduce a bias due to the availability of tumour tissue after cancer diagnosis (see “Statistical analysis” section for details).

^b^The multivariable Cox regression model initially included sex, age, year of diagnosis, family history of colorectal cancer, tumour location, disease stage, tumour differentiation, microsatellite instability, CpG island methylator phenotype, *KRAS* mutation*, BRAF* mutation, *PIK3CA* mutation, long-interspersed nucleotide element-1 methylation level, PTGS2 (cyclooxygenase-2) expression and nuclear CTNNB1 (beta-catenin) expression. A backward elimination with a threshold *P* of 0.05 was used to select variables for the final models.

^c^*P*_trend_ value was calculated across the ordinal categories (negative/low, intermediate and high) of each lymphocytic reaction component in the IPW-adjusted Cox regression model.

As secondary analyses, we examined lymphocytic reaction and patient survival in strata of MSI status or neoantigen load. The prognostic associations of lymphocytic reaction were not significantly modified by either variable (*P*_interaction_ > 0.2 for colorectal cancer-specific survival in strata of MSI status and neoantigen load, Tables 3 and 4).

**Table 3:** Lymphocytic reaction components and patient survival in strata of microsatellite instability (MSI) status.

		Colorectal cancer-specific survival			Overall survival		
	No. of cases	No. of events	Univariable HR (95% CI)^a^	Multivariable HR (95% CI)^a,b^	No. of events	Univariable HR (95% CI)^a^	Multivariable HR (95% CI)^a,b^
Non-MSI-high							
*Crohn’s-like lymphoid reaction*							
Negative/low	721	259	1 (referent)	1 (referent)	452	1 (referent)	1 (referent)
Intermediate	128	27	0.55 (0.36–0.85)	0.56 (0.36–0.87)	70	0.65 (0.47–0.91)	0.67 (0.48–0.94)
High	32	8	0.63 (0.30–1.33)	0.44 (0.17–1.12)	20	0.63 (0.34–1.18)	0.51 (0.25–1.06)
MSI-high							
*Crohn’s-like lymphoid reaction*							
Negative/low	69	10	1 (referent)	1 (referent)	46	1 (referent)	1 (referent)
Intermediate	58	7	0.73 (0.27–2.01)	1.11 (0.38–3.27)	27	0.66 (0.40–1.08)	0.78 (0.46–1.33)
High	49	2	0.17 (0.04–0.82)	0.12 (0.02–0.61)	30	0.48 (0.27–0.83)	0.39 (0.20–0.75)
*P*_interaction_^c^			0.41	0.42		0.82	0.71
Non-MSI-high							
*Peritumoural lymphocytic reaction*							
Negative/low	163	84	1 (referent)	1 (referent)	113	1 (referent)	1 (referent)
Intermediate	796	248	0.54 (0.42–0.71)	0.54 (0.41–0.71)	479	0.54 (0.43–0.69)	0.57 (0.45–0.73)
High	117	24	0.35 (0.21–0.57)	0.31 (0.18–0.56)	67	0.56 (0.39–0.79)	0.48 (0.33–0.72)
MSI-high							
*Peritumoural lymphocytic reaction*							
Negative/low	13	4	1 (referent)	1 (referent)	9	1 (referent)	1 (referent)
Intermediate	120	19	0.59 (0.18–1.91)	0.87 (0.22–3.51)	76	0.56 (0.26–1.23)	0.93 (0.40–2.15)
High	81	4	0.14 (0.03–0.64)	0.21 (0.04–1.14)	44	0.50 (0.22–1.11)	0.71 (0.30–1.65)
*P*_interaction_^c^			0.21	0.63		0.55	0.32
Non-MSI-high							
*Intratumoural perigrandular reaction*							
Negative/low	146	72	1 (referent)	1 (referent)	94	1 (referent)	1 (referent)
Intermediate	846	269	0.56 (0.43–0.74)	0.53 (0.40–0.71)	517	0.61 (0.47–0.78)	0.62 (0.48–0.81)
High	88	16	0.27 (0.15–0.48)	0.20 (0.10–0.41)	49	0.45 (0.30–0.69)	0.37 (0.22–0.60)
MSI-high							
*Intratumoural perigrandular reaction*							
Negative/low	12	3	1 (referent)	1 (referent)	8	1 (referent)	1 (referent)
Intermediate	127	20	0.74 (0.20–2.75)	0.75 (0.15–3.77)	82	0.68 (0.30–1.55)	0.82 (0.29–2.32)
High	75	4	0.18 (0.04–0.87)	0.16 (0.03–1.05)	39	0.48 (0.20–1.14)	0.49 (0.17–1.47)
*P*_interaction_^c^			0.35	0.53		0.72	0.74
Non-MSI-high							
*Tumour-infiltrating lymphocytes*							
Negative/low	889	299	1 (referent)	1 (referent)	548	1 (referent)	1 (referent)
Intermediate	137	47	0.96 (0.69–1.34)	0.78 (0.55–1.11)	81	0.96 (0.72–1.27)	0.81 (0.59–1.10)
High	53	10	0.48 (0.24–0.94)	0.46 (0.21–1.04)	30	0.60 (0.36–1.00)	0.58 (0.31–1.08)
MSI-high							
*Tumour-infiltrating lymphocytes*							
Negative/low	63	13	1 (referent)	1 (referent)	39	1 (referent)	1 (referent)
Intermediate	67	7	0.45 (0.17–1.20)	0.52 (0.17–1.58)	38	0.83 (0.49–1.41)	0.82 (0.43–1.55)
High	84	7	0.36 (0.14–0.95)	0.36 (0.12–1.05)	52	0.77 (0.46–1.26)	0.69 (0.37–1.30)
*P*_interaction_^c^			0.24	0.50		0.88	0.77

*CI* confidence interval, *HR* hazard ratio, *IPW* inverse probability weighting, *MSI* microsatellite instability.

^a^IPW was applied to reduce a bias due to the availability of tumour tissue after cancer diagnosis (see “Statistical analysis” section for details).

^b^The multivariable Cox regression model initially included sex, age, year of diagnosis, family history of colorectal cancer, tumour location, disease stage, tumour differentiation, CpG island methylator phenotype, *KRAS* mutation*, BRAF* mutation, *PIK3CA* mutation, long-interspersed nucleotide element-1 methylation level, PTGS2 (cyclooxygenase-2) expression and nuclear CTNNB1 (beta-catenin) expression. A backward elimination with a threshold *P* of 0.05 was used to select variables for the final models.

^c^*P*_interaction_ value (two-sided) was calculated using the Wald test for the cross-product of the ordinal category (negative/low, intermediate and high) of each lymphocytic reaction component and MSI status (non-high vs. high) in the IPW-adjusted Cox regression model.

**Table 4:** Lymphocytic reaction components and patient survival in strata of tumour neoantigen load.

		Colorectal cancer-specific survival			Overall survival		
	No. of cases	No. of events	Univariable HR (95% CI)^a^	Multivariable HR (95% CI)^a,b^	No. of events	Univariable HR (95% CI)^a^	Multivariable HR (95% CI)^a,b^
Neoantigen-low							
*Crohn’s-like lymphoid reaction*							
Negative/low	205	66	1 (referent)	1 (referent)	117	1 (referent)	1 (referent)
Intermediate/high	43	9	0.57 (0.28–1.16)	0.63 (0.29–1.39)	22	0.61 (0.36–1.03)	0.68 (0.38–1.22)
Neoantigen-high							
*Crohn’s-like lymphoid reaction*							
Negative/low	174	53	1 (referent)	1 (referent)	111	1 (referent)	1 (referent)
Intermediate/high	83	11	0.35 (0.18–0.68)	0.54 (0.26–1.11)	41	0.55 (0.37–0.84)	0.58 (0.38–0.89)
*P*_interaction_^c^			0.31	0.29		0.89	0.85
Neoantigen-low							
*Peritumoural lymphocytic reaction*							
Negative/low	55	22	1 (referent)	1 (referent)	33	1 (referent)	1 (referent)
Intermediate/high	232	65	0.57 (0.35–0.94)	0.72 (0.43–1.21)	128	0.61 (0.40–0.93)	0.68 (0.45–1.02)
Neoantigen-high							
*Peritumoural lymphocytic reaction*							
Negative/low	42	22	1 (referent)	1 (referent)	32	1 (referent)	1 (referent)
Intermediate/high	245	47	0.34 (0.19–0.60)	0.35 (0.20–0.62)	135	0.45 (0.28–0.73)	0.52 (0.33–0.81)
*P*_interaction_^c^			0.090	0.23		0.47	0.73
Neoantigen-low							
*Intratumoural perigrandular reaction*							
Negative/low	50	22	1 (referent)	1 (referent)	30	1 (referent)	1 (referent)
Intermediate/high	238	65	0.50 (0.31–0.80)	0.45 (0.29–0.72)	131	0.61 (0.40–0.94)	0.59 (0.39–0.90)
Neoantigen-high							
*Intratumoural perigrandular reaction*							
Negative/low	40	20	1 (referent)	1 (referent)	27	1 (referent)	1 (referent)
Intermediate/high	247	49	0.32 (0.18–0.56)	0.32 (0.19–0.57)	140	0.48 (0.30–0.76)	0.50 (0.31–0.81)
*P*_interaction_^c^			0.95	0.56		0.85	0.23
Neoantigen-low							
*Tumour-infiltrating lymphocytes*							
Negative/low	239	75	1 (referent)	1 (referent)	138	1 (referent)	1 (referent)
Intermediate/high	49	12	0.69 (0.37–1.30)	0.64 (0.32–1.25)	23	0.58 (0.33–0.99)	0.52 (0.28–0.98)
Neoantigen-high							
*Tumour-infiltrating lymphocytes*							
Negative/low	163	50	1 (referent)	1 (referent)	101	1 (referent)	1 (referent)
Intermediate/high	122	18	0.38 (0.21–0.67)	0.42 (0.22–0.80)	65	0.64 (0.44–0.93)	0.60 (0.41–0.89)
*P*_interaction_^c^			0.62	0.67		0.089	0.052

*CI* confidence interval, *HR* hazard ratio, *IPW* inverse probability weighting.

^a^IPW was applied to reduce a bias due to the availability of tumour tissue after cancer diagnosis (see “Statistical analysis” section for details).

^b^The multivariable Cox regression model initially included sex, age, year of diagnosis, family history of colorectal cancer, tumour location, disease stage, tumour differentiation, microsatellite instability, CpG island methylator phenotype, *KRAS* mutation*, BRAF* mutation, *PIK3CA* mutation, long-interspersed nucleotide element-1 methylation level, PTGS2 (cyclooxygenase-2) expression and nuclear CTNNB1 (beta-catenin) expression. A backward elimination with a threshold *P* of 0.05 was used to select variables for the final models.

^c^*P*_interaction_ value (two-sided) was calculated using the Wald test for the cross-product of the ordinal category (negative/low, intermediate and high) of each lymphocytic reaction component and tumour neoantigen loads (continuous, log-transformed) in the IPW-adjusted Cox regression model.

We also examined patient survival according to the overall lymphocytic reaction score. In multivariable Cox regression analyses, a higher overall lymphocytic reaction score was associated with better colorectal cancer-specific survival and overall survival (*P*_trend_ ≤ 0.0001 for both, Supplementary Table S4). The Cox regression analyses without IPW yielded similar results to the IPW-adjusted model (Supplementary Table S5). When we adjusted for neoantigen load as well as MSI, these findings remained unchanged (*P*_trend_ = 0.0048 for cancer-specific survival and *P*_trend_ = 0.0016 for overall survival, Supplementary Table S6). In Kaplan–Meier survival analyses, the overall lymphocytic reaction score was positively associated with favourable colorectal cancer-specific survival (*P* < 0.0001 by the log-rank test for trend, Supplementary Fig. S1).

As another secondary analysis, given the advance in the treatment strategy over the decades, we assessed the prognostic association of lymphocytic reaction in strata of the year of diagnosis and tumour location. The prognostic associations of lymphocytic reaction were not significantly modified by either variable (*P*_interaction_ > 0.1 for colorectal cancer-specific survival in strata of year of diagnosis and tumour location, Supplementary Tables S7 and S8).

As exploratory analyses, we assessed the prognostic interactions between the lymphocytic reaction components in relation to colorectal cancer-specific mortality. There was no prognostic interaction between the lymphocytic reaction components (*P*_interaction_ > 0.1) (Supplementary Tables S9 and S10).

## Discussion

Utilising two US prospective cohort studies, we found that higher levels of each of four lymphocytic reaction components, and higher overall lymphocytic reaction score, were strongly associated with better colorectal cancer survival. Notably, these prognostic associations were not significantly modified by adjusting for potential confounders, including MSI, CIMP*, BRAF* mutation, LINE-1 methylation and neoantigen load. These findings provide strong population-based evidence for the role of host immunity in colorectal cancer prognosis. Since lymphocytic reaction can be examined by evaluating haematoxylin- and eosin-stained tissues, our study also supports the potential of lymphocytic reaction as a prognostic marker for colorectal cancer patients that could be readily implemented in clinical work.

Lymphocytic reaction has been demonstrated to reflect local immune effector response in colorectal cancer, associated with patient survival.^6–8,34–37^ The assessment of the host immunity might also be helpful to advance current front-line immunotherapies, as immune checkpoint inhibitors aim to reactivate T-cell-mediated antitumour immune response.^38–40^ Evidence suggests that not only abundance but also spatial localisation of immune cells is prognostically relevant.^34–37^ Our previous study using a population of 843 colorectal cancer patients has shown a significant positive association of lymphocytic reaction with favourable patient survival independent of tumour molecular characteristics, including CIMP, MSI status and LINE-1 hypomethylation.^5^ Specifically, this association was most robust when using the overall lymphocytic score, while the four lymphocytic reaction components (Crohn’s-like lymphoid reaction, peritumoural lymphocytic reaction, intratumoural periglandular reaction and TIL) had weaker associations with survival. This supported the value of grading different lymphocytic reaction components to generate a composite lymphocytic reaction score. Few other studies have evaluated the prognostic significance of such composite score, but some have reported that the individual components of the lymphocytic reaction, including TIL and Crohn’s-like lymphoid reaction, are independently associated with lower colorectal cancer mortality after adjustment for MSI status.^7,8^ In this study, with an expanded sample size (1465 cases) and additional important potential confounders (*PIK3CA* mutation, PTGS2 expression, nuclear CTNNB1 expression and neoantigen load), we identified a significant association of each of the four lymphocytic reaction components with colorectal cancer-specific survival independent of the potential confounders. In addition, only a few studies, including ours, evaluated “true” TIL that exists on top of tumour epithelium,^5,7^ whereas most studies have not distinguished lymphocytes in tumour stromal regions (intratumoural periglandular reaction) from the true TIL. Thus, this study supports the robust prognostic value of both the overall lymphocytic reaction score and its four components, suggesting that the comprehensive characterisation of the lymphocytic infiltrate in different areal regions provides valuable information about the host antitumour immune response. Finally, IPW was used to minimise the potential selection bias caused by biospecimen availability.^19–22^ The IPW method can utilise the information from all the incident 4420 colorectal cancer cases within the cohorts during the follow-up period in order to produce less-biased estimation of the prognostic association of lymphocytic reaction. The differences of the results between IPW-applied analysis and analysis without IPW were minor, which suggests that the selection bias may not be a major concern in this dataset, and supports the robustness of our current analyses.

Colorectal cancer represents a heterogeneous group of tumours that result from not only a progressive accumulation of somatic molecular alterations, but also various host–tumour interactions, including antitumour immunity.^41–43^ The assessment of host immunity against colorectal cancer in the tumour microenvironment is increasingly important in the translational research, and biomarkers representing tumour molecular characteristics and the immune microenvironment are likely to be more and more included in the future tumour pathology evaluation criteria.^6,35,44^ Thus, integrated analyses of the immune response and tumour molecular features are necessary for the development of new immune biomarkers. In this study, we have included important confounders, including MSI, CIMP, LINE-1 methylation, *KRAS*, *BRAF* and *PIK3CA* mutation, PTGS2 expression, nuclear CTNNB1 expression and neoantigen load. Neoantigens are the most interesting targets for immunotherapies since neoepitopes are not subject to central tolerance in the thymus.^45^ Peptides of neoantigens bound to HLA can be recognised by T cells, which initiate antitumour immune response. Our study further supports the finding that neoantigen load is positively associated with higher lymphocytic reactions in colorectal cancer patients. Importantly, the benefit associated with higher lymphocytic reaction was not significantly modified by the neoantigen load and other molecular features, confirming the independent role of lymphocytic reaction in colorectal cancer survival. To the best of our knowledge, there has been no previous study on lymphocytic reaction and patient survival, which has controlled as many molecular variables as we did in this study.

We need to point out several limitations in our study. First, there is limited data on cancer treatments in our study cohort. However, it was unlikely that clinical treatment decisions were influenced by lymphocytic reaction, because these data were not available to treating physicians. In addition, given the advances in colorectal cancer treatment, as well as differences between treatment strategies of colon and rectal carcinoma, we conducted stratified analyses according to year of diagnosis and tumour location. Second, data on cancer recurrences were not collected. However, colorectal cancer-specific mortality is considered as a reasonable colorectal cancer-specific outcome, since these two cohorts had a long follow-up duration of censored cases. Third, our study was based on evaluation of immune cells by haematoxylin- and eosin-stained tissue sections. Accumulating evidence suggests that specific immune cell types are differentially involved in host immune response.^34–37,46–48^ Innate immune response also plays a crucial role in the tumour immune microenvironment, and may interact with adaptive immune cells.^40^ Further identification of these immune cell types and immunoregulatory molecules, driving each component of the lymphocytic reaction, could contribute to better understanding of the tumour immune microenvironment. Finally, we had limited information of tumour pathological features in this study. Pathological features, such as lympho-vascular invasion, extramural vascular invasion, perineural invasion and tumour budding, represent potential unmeasured confounding factors of the current analyses.^49–51^

The strengths of our study include utilising the two independent US prospective cohorts, which covered data on pathological findings and tumour molecular features.^12,52^ This population-based colorectal cancer database enabled us to rigorously examine the interactive prognostic value of lymphocytic reaction and each of lymphocytic reaction components, controlling for potential confounders. The molecular pathological epidemiology method has been utilised to assess the combined influences of exposures and immunity in cancer. In addition, compared with our previous study, an increased number of cases allow us to control for a larger group of confounders, and we utilised the IPW method to reduce the potential bias by the availability of colorectal cancer tissue.

In conclusion, a higher overall lymphocytic reaction score, along with four lymphocytic reaction components, is strongly associated with better colorectal cancer-specific survival, independent of MSI status, neoantigen load and other tumour and patient characteristics. Our population-based data support the role of host immune response as an independent prognostic indicator in colorectal cancer.

## Use of standardised official symbols

We use HUGO (Human Genome Organisation)-approved official symbols (or root symbols) for genes and gene products, including BRAF, CACNA1G, CD274, CDKN2A, CRABP1, CTNNB1, IGF2, KRAS, MLH1, NEUROG1, PDCD1, PIK3CA, PTGS2, RUNX3, and SOCS1; all of which are described at www.genenames.org. Gene symbols are italicised whereas symbols for gene products are not italicised.

## Supplementary information

Supplemental tables and figure

## Data Availability

The data sets used and/or analysed during this study are available from the corresponding author on reasonable request.
