# Supplementary material for: An integrated analysis of lymphocytic reaction, tumour molecular characteristics and patient survival in colorectal cancer
Source: Br J Cancer. 2020 Mar 11;122(9):1367–77. doi: 10.1038/s41416-020-0780-3 (PMC7188805; doi:10.1038/s41416-020-0780-3)

Supplementary Table S1. Clinical, pathological, and molecular characteristics of colorectal cancer cases according to the Crohn-like lymphoid reaction and peritumoural lymphocytic reaction to colorectal cancer

|  |  | Crohn’s-like lymphoid reaction | | |  |  |  | Peritumoural lymphocytic reaction | | |  |
| --- | --- | --- | --- | --- | --- | --- | --- | --- | --- | --- | --- |
| Characteristic^1^ | No. of cases  (N = 1,195) | Negative/low  (N = 903) | Intermediate  (N = 205) | High  (N = 87) | *P* value^2^ |  | No. of cases  (N = 1,456) | Negative/low  (N = 210) | Intermediate  (N = 1,022) | High  (N = 224) | *P* value^2^ |
|  |  |  |  |  |  |  |  |  |  |  |  |
| Sex |  |  |  |  | 0.75 |  |  |  |  |  | 0.87 |
| Female (NHS) | 681 (57%) | 516 (57%) | 113 (55%) | 52 (60%) |  |  | 818 (56%) | 119 (57%) | 570 (56%) | 129 (58%) |  |
| Male (HPFS) | 514 (43%) | 387 (43%) | 92 (45%) | 35 (40%) |  |  | 638 (44%) | 91 (43%) | 452 (44%) | 95 (42%) |  |
|  |  |  |  |  |  |  |  |  |  |  |  |
| Mean age ± SD (years) | 69.3 ± 9.0 | 69.0 ± 9.1 | 69.9 ± 8.8 | 71.3 ± 7.7 | 0.059 |  | 69.0 ± 9.0 | 70.9 ± 9.3 | 67.9 ± 8.9 | 72.2 ± 8.1 | < 0.0001 |
|  |  |  |  |  |  |  |  |  |  |  |  |
| Year of diagnosis |  |  |  |  | 0.027 |  |  |  |  |  | < 0.0001 |
| 1995 or before | 415 (35%) | 326 (36%) | 63 (31%) | 26 (30%) |  |  | 520 (36%) | 40 (19%) | 441 (43%) | 39 (17%) |  |
| 1996-2000 | 322 (27%) | 233 (26%) | 54 (26%) | 35 (40%) |  |  | 440 (30%) | 49 (23%) | 330 (32%) | 61 (27%) |  |
| 2001-2008 | 458 (38%) | 344 (38%) | 88 (43%) | 26 (30%) |  |  | 496 (34%) | 121 (58%) | 251 (25%) | 124 (55%) |  |
|  |  |  |  |  |  |  |  |  |  |  |  |
| Family history of colorectal  cancer in first-degree  relative(s) |  |  |  |  | 0.69 |  |  |  |  |  | 0.50 |
| Absent | 959 (81%) | 728 (81%) | 164 (81%) | 67 (77%) |  |  | 1,168 (80%) | 169 (80%) | 825 (81%) | 174 (78%) |  |
| Present | 232 (19%) | 173 (19%) | 39 (19%) | 20 (23%) |  |  | 283 (20%) | 41 (20%) | 192 (19%) | 50 (22%) |  |
|  |  |  |  |  |  |  |  |  |  |  |  |
| Tumour location |  |  |  |  | < 0.0001 |  |  |  |  |  | < 0.0001 |
| Cecum | 204 (17%) | 141 (16%) | 44 (22%) | 19 (22%) |  |  | 255 (18%) | 25 (12%) | 185 (18%) | 45 (20%) |  |
| Ascending to transverse  colon | 384 (32%) | 252 (28%) | 92 (45%) | 40 (46%) |  |  | 447 (31%) | 68 (33%) | 284 (28%) | 95 (43%) |  |
| Descending to sigmoid  colon | 353 (30%) | 286 (32%) | 47 (23%) | 20 (23%) |  |  | 436 (30%) | 67 (32%) | 316 (31%) | 53 (24%) |  |
| Rectum | 250 (21%) | 221 (25%) | 21 (10%) | 8 (9.2%) |  |  | 312 (22%) | 47 (23%) | 235 (23%) | 30 (13%) |  |
|  |  |  |  |  |  |  |  |  |  |  |  |
| AJCC disease stage |  |  |  |  | < 0.0001 |  |  |  |  |  | < 0.0001 |
| I | 275 (25%) | 212 (26%) | 41 (21%) | 22 (26%) |  |  | 342 (26%) | 30 (16%) | 241 (26%) | 71 (34%) |  |
| II | 376 (34%) | 240 (29%) | 98 (49%) | 38 (45%) |  |  | 426 (32%) | 49 (26%) | 299 (32%) | 78 (37%) |  |
| III | 316 (28%) | 245 (29%) | 50 (25%) | 21 (25%) |  |  | 371 (28%) | 55 (29%) | 265 (29%) | 51 (24%) |  |
| IV | 147 (13%) | 134 (16%) | 9 (4.6%) | 4 (4.7%) |  |  | 184 (14%) | 54 (29%) | 121 (13%) | 9 (4.3%) |  |
|  |  |  |  |  |  |  |  |  |  |  |  |
| Tumour differentiation |  |  |  |  | < 0.0001 |  |  |  |  |  | < 0.0001 |
| Well to moderate | 1,072 (91%) | 834 (94%) | 179 (87%) | 59 (68%) |  |  | 1,293 (90%) | 186 (89%) | 931 (92%) | 176 (79%) |  |
| Poor | 112(9.5%) | 58 (6.5%) | 26 (13%) | 28 (32%) |  |  | 151 (10%) | 23 (11%) | 80 (7.9%) | 48 (21%) |  |
|  |  |  |  |  |  |  |  |  |  |  |  |
| MSI status |  |  |  |  | < 0.0001 |  |  |  |  |  | < 0.0001 |
| Non-MSI-high | 881 (83%) | 721 (91%) | 128 (69%) | 32 (40%) |  |  | 881 (83%) | 154 (92%) | 627 (87%) | 100 (59%) |  |
| MSI-high | 176 (17%) | 69 (8.7%) | 58 (31%) | 49 (60%) |  |  | 176 (17%) | 13 (7.8%) | 94 (13%) | 69 (41%) |  |
|  |  |  |  |  |  |  |  |  |  |  |  |
| CIMP status |  |  |  |  | < 0.0001 |  |  |  |  |  | < 0.0001 |
| Low/negative | 832 (82%) | 681 (89%) | 118 (68%) | 33 (42%) |  |  | 832 (82%) | 142 (88%) | 602 (86%) | 88 (58%) |  |
| High | 183 (18%) | 82 (11%) | 55 (32%) | 46 (58%) |  |  | 183 (18%) | 19 (12%) | 100 (14%) | 64 (42%) |  |
|  |  |  |  |  |  |  |  |  |  |  |  |
| Mean LINE-1 methylation  level ± SD (%) | 63.8 ± 9.9 | 63.1 ± 9.8 | 64.7 ± 9.5 | 67.8 ± 10.4 | 0.0001 |  | 63.2 ± 9.9 | 63.5 ± 10.4 | 62.4 ± 9.5 | 66.7 ± 10.6 | < 0.0001 |
|  |  |  |  |  |  |  |  |  |  |  |  |
| *KRAS* mutation |  |  |  |  | 0.030 |  |  |  |  |  | 0.25 |
| Wild-type | 586 (58%) | 423 (56%) | 111 (63%) | 52 (68%) |  |  | 586 (58%) | 91 (57%) | 398 (57%) | 97 (64%) |  |
| Mutant | 423 (42%) | 335 (44%) | 64 (37%) | 24 (32%) |  |  | 423 (42%) | 68 (43%) | 301 (43%) | 54 (36%) |  |
|  |  |  |  |  |  |  |  |  |  |  |  |
| *BRAF* mutation |  |  |  |  | < 0.0001 |  |  |  |  |  | < 0.0001 |
| Wild-type | 893 (84%) | 707 (89%) | 138 (75%) | 48 (60%) |  |  | 893 (84%) | 145 (87%) | 626 (86%) | 122 (73%) |  |
| Mutant | 167 (16%) | 89 (11%) | 46 (25%) | 32 (40%) |  |  | 167 (16%) | 22 (13%) | 99 (14%) | 46 (27%) |  |
|  |  |  |  |  |  |  |  |  |  |  |  |
| *PIK3CA* mutation |  |  |  |  | 0.95 |  |  |  |  |  | 0.61 |
| Wild-type | 829 (84%) | 627 (84%) | 140 (83%) | 62 (85%) |  |  | 829 (84%) | 132 (81%) | 567 (85%) | 130 (83%) |  |
| Mutant | 161 (16%) | 122 (16%) | 28 (17%) | 11 (15%) |  |  | 161 (16%) | 30 (19%) | 104 (15%) | 27 (17%) |  |
|  |  |  |  |  |  |  |  |  |  |  |  |
| CD274 (PD-L1) expression  score |  |  |  |  | 0.17 |  |  |  |  |  | 0.071 |
| 0 | 69 (10%) | 46 (9.1%) | 14 (11%) | 9 (19%) |  |  | 69 (10%) | 7 (6.0%) | 46 (10%) | 16 (15%) |  |
| 1 | 195 (29%) | 146 (29%) | 34 (27%) | 15 (32%) |  |  | 195 (29%) | 35 (30%) | 127 (28%) | 33 (31%) |  |
| 2 | 192 (28%) | 136 (27%) | 44 (35%) | 12 (26%) |  |  | 192 (28%) | 42 (36%) | 120 (26%) | 30 (28%) |  |
| 3 | 192 (28%) | 153 (30%) | 31 (24%) | 8 (17%) |  |  | 192 (28%) | 26 (22%) | 145 (32%) | 21 (20%) |  |
| 4 | 33 (4.9%) | 26 (5.1%) | 4 (3.2%) | 3 (6.4%) |  |  | 33 (4.9%) | 6 (5.2%) | 21 (4.6%) | 6 (5.7%) |  |
|  |  |  |  |  |  |  |  |  |  |  |  |
| PTGS2 (cyclooxygenase-2)  expression |  |  |  |  | 0.13 |  |  |  |  |  | 0.088 |
| Negative | 377 (38%) | 271 (37%) | 70 (40%) | 36 (48%) |  |  | 377 (38%) | 61 (40%) | 250 (36%) | 66 (46%) |  |
| Positive | 613 (62%) | 469 (63%) | 105 (60%) | 39 (52%) |  |  | 613 (62%) | 91 (60%) | 443 (64%) | 79 (54%) |  |
|  |  |  |  |  |  |  |  |  |  |  |  |
| Nuclear CTNNB1  (beta-catenin) expression |  |  |  |  | 0.0043 |  |  |  |  |  | 0.0020 |
| Negative | 765 (64%) | 560 (62%) | 136 (66%) | 69 (79%) |  |  | 935 (64%) | 133 (63%) | 635 (62%) | 167 (75%) |  |
| Positive | 430 (36%) | 343 (38%) | 69 (34%) | 18 (21%) |  |  | 521 (36%) | 77 (37%) | 387 (38%) | 57 (25%) |  |
|  |  |  |  |  |  |  |  |  |  |  |  |
| Neoantigen load |  |  |  |  | < 0.0001 |  |  |  |  |  | 0.0046 |
| Q1 (lowest) | 125 (25%) | 103 (27%) | 16 (19%) | 6 (14%) |  |  | 125 (25%) | 31 (33%) | 71 (23%) | 23 (21%) |  |
| Q2 | 123 (24%) | 102 (27%) | 16 (19%) | 5 (12%) |  |  | 123 (24%) | 22 (24%) | 80 (26%) | 21 (19%) |  |
| Q3 | 129 (26%) | 111 (29%) | 13 (15%) | 5 (12%) |  |  | 129 (26%) | 28 (30%) | 77 (25%) | 24 (22%) |  |
| Q4 (highest) | 128 (25%) | 63 (17%) | 39 (46%) | 26 (62%) |  |  | 128 (25%) | 12 (13%) | 75 (25%) | 41 (38%) |  |
|  |  |  |  |  |  |  |  |  |  |  |  |

^1^ Percentage indicates the proportion of patients with a specific clinical, pathological, or molecular characteristic among all patients or in strata of lymphocytic reaction to colorectal cancer.

^2^ To assess associations between the ordinal categories (negative/low, intermediate and high) of Crohn’s-like lymphoid reaction or peritumoural lymphocytic reaction to colorectal cancer, and categorical data, the chi-square test was performed. To compare age, and LINE-1 methylation level, an analysis of variance was performed.

Abbreviations: AJCC, American Joint Committee on Cancer; CIMP, CpG island methylator phenotype; HPFS, Health Professionals Follow-up Study; LINE-1, long-interspersed nucleotide element-1; MSI, microsatellite instability; NHS, Nurses’ Health Study; SD, standard deviation.

Supplementary Table S2. Lymphocytic reaction components and patient survival without inverse probability weighting

|  |  | Colorectal cancer-specific survival | | |  | Overall survival | | |
| --- | --- | --- | --- | --- | --- | --- | --- | --- |
|  | No. of  cases | No. of  events | Univariable  HR (95% CI) | Multivariable  HR (95% CI)^1^ |  | No. of  events | Univariable  HR (95% CI) | Multivariable  HR (95% CI)^1^ |
|  |  |  |  |  |  |  |  |  |
| Crohn’s-like lymphoid reaction |  |  |  |  |  |  |  |  |
| Negative/low | 903 | 305 | 1 (referent) | 1 (referent) |  | 563 | 1 (referent) | 1 (referent) |
| Intermediate | 205 | 37 | 0.48 (0.34-0.68) | 0.56 (0.40-0.79) |  | 108 | 0.68 (0.53-0.86) | 0.73 (0.57-0.93) |
| High | 87 | 10 | 0.29 (0.15-0.54) | 0.29 (0.15-0.56) |  | 54 | 0.57 (0.40-0.83) | 0.57 (0.39-0.84) |
| *P*_trend_^2^ |  |  | < 0.0001 | < 0.0001 |  |  | < 0.0001 | < 0.0001 |
|  |  |  |  |  |  |  |  |  |
| Peritumoural lymphocytic reaction |  |  |  |  |  |  |  |  |
| Negative/low | 210 | 107 | 1 (referent) | 1 (referent) |  | 146 | 1 (referent) | 1 (referent) |
| Intermediate | 1,022 | 290 | 0.44 (0.35-0.55) | 0.48 (0.38-0.61) |  | 611 | 0.49 (0.40-0.59) | 0.57 (0.46-0.70) |
| High | 224 | 32 | 0.22 (0.15-0.33) | 0.27 (0.18-0.40) |  | 124 | 0.49 (0.37-0.63) | 0.51 (0.39-0.67) |
| *P*_trend_^2^ |  |  | < 0.0001 | < 0.0001 |  |  | < 0.0001 | < 0.0001 |
|  |  |  |  |  |  |  |  |  |
| Intratumoural perigrandular reaction |  |  |  |  |  |  |  |  |
| Negative/low | 193 | 91 | 1 (referent) | 1 (referent) |  | 124 | 1 (referent) | 1 (referent) |
| Intermediate | 1,085 | 315 | 0.51 (0.40-0.64) | 0.53 (0.42-0.68) |  | 661 | 0.59 (0.48-0.73) | 0.67 (0.54-0.83) |
| High | 184 | 24 | 0.22 (0.14-0.34) | 0.23 (0.15-0.37) |  | 98 | 0.45 (0.33-0.62) | 0.46 (0.34-0.63) |
| *P*_trend_^2^ |  |  | < 0.0001 | < 0.0001 |  |  | < 0.0001 | < 0.0001 |
|  |  |  |  |  |  |  |  |  |
| Tumour-infiltrating lymphocytes |  |  |  |  |  |  |  |  |
| Negative/low | 1,095 | 356 | 1 (referent) | 1 (referent) |  | 670 | 1 (referent) | 1 (referent) |
| Intermediate | 219 | 57 | 0.79 (0.60-1.04) | 0.74 (0.56-0.99) |  | 126 | 0.94 (0.76-1.18) | 0.88 (0.70-1.09) |
| High | 147 | 17 | 0.32 (0.20-0.52) | 0.38 (0.23-0.63) |  | 87 | 0.67 (0.51-0.89) | 0.66 (0.48-0.90) |
| *P*_trend_^2^ |  |  | < 0.0001 | 0.0020 |  |  | 0.010 | 0.0049 |
|  |  |  |  |  |  |  |  |  |

^1^ The multivariable Cox regression model initially included sex, age, year of diagnosis, family history of colorectal cancer, tumour location, disease stage, tumour differentiation, microsatellite instability, CpG island methylator phenotype, *KRAS* mutation*, BRAF* mutation, *PIK3CA* mutation, long-interspersed nucleotide element-1 methylation level, PTGS2 (cyclooxygenase-2) expression, and nuclear CTNNB1 (beta-catenin) expression. A backward elimination with a threshold *P* of 0.05 was used to select variables for the final models.

^2^ *P*_trend_ value was calculated across the ordinal categories (negative/low, intermediate, and high) of each lymphocytic reaction component in the Cox regression model.

Abbreviations: CI, confidence interval; HR, hazard ratio.

Supplementary Table S3. Lymphocytic reaction components and patient survival in colorectal cancer cases with neoantigen load data

|  |  | Colorectal cancer-specific survival | | |  | Overall survival | | |
| --- | --- | --- | --- | --- | --- | --- | --- | --- |
|  | No. of  cases | No. of  events | Univariable  HR (95% CI)^1^ | Multivariable  HR (95% CI)^1,2^ |  | No. of  events | Univariable  HR (95% CI)^1^ | Multivariable  HR (95% CI)^1,2^ |
|  |  |  |  |  |  |  |  |  |
| Crohn’s-like lymphoid reaction |  |  |  |  |  |  |  |  |
| Negative/low | 379 | 119 | 1 (referent) | 1 (referent) |  | 228 | 1 (referent) | 1 (referent) |
| Intermediate | 84 | 15 | 0.46 (0.27-0.79) | 0.64 (0.36-1.15) |  | 39 | 0.53 (0.36-0.78) | 0.57 (0.38-0.86) |
| High | 42 | 5 | 0.35 (0.13-0.92) | 0.47 (0.16-1.36) |  | 24 | 0.65 (0.40-1.06) | 0.66 (0.40-1.09) |
| *P*_trend_^3^ |  |  | 0.0023 | 0.095 |  |  | 0.0055 | 0.015 |
|  |  |  |  |  |  |  |  |  |
| Peritumoural lymphocytic reaction |  |  |  |  |  |  |  |  |
| Negative/low | 97 | 44 | 1 (referent) | 1 (referent) |  | 65 | 1 (referent) | 1 (referent) |
| Intermediate | 359 | 95 | 0.51 (0.35-0.74) | 0.55 (0.37-0.81) |  | 205 | 0.53 (0.38-0.74) | 0.60 (0.44-0.83) |
| High | 118 | 17 | 0.28 (0.16-0.51) | 0.43 (0.23-0.80) |  | 58 | 0.54 (0.36-0.79) | 0.58 (0.39-0.86) |
| *P*_trend_^3^ |  |  | < 0.0001 | 0.0018 |  |  | 0.0031 | 0.0076 |
|  |  |  |  |  |  |  |  |  |
| Intratumoural perigrandular reaction |  |  |  |  |  |  |  |  |
| Negative/low | 90 | 42 | 1 (referent) | 1 (referent) |  | 57 | 1 (referent) | 1 (referent) |
| Intermediate | 401 | 104 | 0.45 (0.31-0.65) | 0.40 (0.28-0.57) |  | 234 | 0.58 (0.42-0.80) | 0.57 (0.42-0.78) |
| High | 84 | 10 | 0.20 (0.09-0.41) | 0.24 (0.11-0.54) |  | 37 | 0.38 (0.23-0.62) | 0.40 (0.23-0.69) |
| *P*_trend_^3^ |  |  | < 0.0001 | < 0.0001 |  |  | < 0.0001 | 0.0002 |
|  |  |  |  |  |  |  |  |  |
| Tumour-infiltrating lymphocytes |  |  |  |  |  |  |  |  |
| Negative/low | 402 | 125 | 1 (referent) | 1 (referent) |  | 239 | 1 (referent) | 1 (referent) |
| Intermediate | 105 | 23 | 0.61 (0.38-0.97) | 0.58 (0.34-0.98) |  | 55 | 0.70 (0.49-0.99) | 0.63 (0.44-0.92) |
| High | 66 | 7 | 0.26 (0.12-0.56) | 0.25 (0.10-0.63) |  | 33 | 0.50 (0.32-0.77) | 0.44 (0.25-0.76) |
| *P*_trend_^3^ |  |  | < 0.0001 | 0.0006 |  |  | 0.0005 | 0.0002 |
|  |  |  |  |  |  |  |  |  |

^1^ IPW was applied to reduce a bias due to the availability of tumour tissue after cancer diagnosis (see “Statistical analysis” subsection for details).

^2^ The multivariable Cox regression model initially included sex, age, year of diagnosis, family history of colorectal cancer, tumour location, disease stage, tumour differentiation, microsatellite instability, CpG island methylator phenotype, *KRAS* mutation*, BRAF* mutation, *PIK3CA* mutation, long-interspersed nucleotide element-1 methylation level, PTGS2 (cyclooxygenase-2) expression, nuclear CTNNB1 (beta-catenin) expression, and neoantigen load. A backward elimination with a threshold *P* of 0.05 was used to select variables for the final models.

^3^ *P*_trend_ value was calculated across the ordinal categories (negative/low, intermediate, and high) of each lymphocytic reaction component in the IPW-adjusted Cox regression model.

Abbreviations: CI, confidence interval; HR, hazard ratio; IPW, inverse probability weighting.

Supplementary Table S4. Overall lymphocytic reaction score and patient survival

|  |  | Colorectal cancer-specific survival | | |  | Overall survival | | |
| --- | --- | --- | --- | --- | --- | --- | --- | --- |
|  | No. of  cases | No. of  events | Univariable  HR (95% CI)^1^ | Multivariable  HR (95% CI)^1,2^ |  | No. of  events | Univariable  HR (95% CI)^1^ | Multivariable  HR (95% CI)^1,2^ |
|  |  |  |  |  |  |  |  |  |
| Overall lymphocytic reaction score |  |  |  |  |  |  |  |  |
| Low (0-2) | 748 | 270 | 1 (referent) | 1 (referent) |  | 478 | 1 (referent) | 1 (referent) |
| Intermediate (3-6) | 362 | 71 | 0.48 (0.36-0.63) | 0.54 (0.40-0.73) |  | 192 | 0.70 (0.57-0.85) | 0.69 (0.56-0.86) |
| High (7-12) | 83 | 9 | 0.26 (0.12-0.54) | 0.29 (0.12-0.68) |  | 53 | 0.62 (0.43-0.89) | 0.58 (0.37-0.90) |
| *P*_trend_^3^ |  |  | < 0.0001 | < 0.0001 |  |  | 0.0001 | 0.0001 |
|  |  |  |  |  |  |  |  |  |

^1^ IPW was applied to reduce a bias due to the availability of tumour tissue after cancer diagnosis (see “Statistical analysis” subsection for details).

^2^ The multivariable Cox regression model initially included sex, age, year of diagnosis, family history of colorectal cancer, tumour location, disease stage, tumour differentiation, microsatellite instability, CpG island methylator phenotype, *KRAS* mutation*, BRAF* mutation, *PIK3CA* mutation, long-interspersed nucleotide element-1 methylation level, PTGS2 (cyclooxygenase-2) expression, and nuclear CTNNB1 (beta-catenin) expression. A backward elimination with a threshold *P* of 0.05 was used to select variables for the final models.

^3^ *P*_trend_ value was calculated across the ordinal categories (low, intermediate, and high) of overall lymphocytic reaction score in the IPW-adjusted Cox regression model.

Abbreviations: CI, confidence interval; HR, hazard ratio; IPW, inverse probability weighting

Supplementary Table S5. Overall lymphocytic reaction score and patient survival without inverse probability Weighting

|  |  | Colorectal cancer-specific survival | | |  | Overall survival | | |
| --- | --- | --- | --- | --- | --- | --- | --- | --- |
|  | No. of  cases | No. of  events | Univariable  HR (95% CI) | Multivariable  HR (95% CI)^1^ |  | No. of  events | Univariable  HR (95% CI) | Multivariable  HR (95% CI)^1^ |
|  |  |  |  |  |  |  |  |  |
| Overall lymphocytic reaction score |  |  |  |  |  |  |  |  |
| Low (0-2) | 748 | 270 | 1 (referent) | 1 (referent) |  | 478 | 1 (referent) | 1 (referent) |
| Intermediate (3-6) | 362 | 71 | 0.50 (0.38-0.65) | 0.55 (0.42-0.72) |  | 192 | 0.73 (0.60-0.88) | 0.71 (0.59-0.86) |
| High (7-12) | 83 | 9 | 0.26 (0.14-0.51) | 0.30 (0.15-0.59) |  | 53 | 0.61 (0.42-0.88) | 0.60 (0.41-0.87) |
| *P*_trend_^2^ |  |  | < 0.0001 | < 0.0001 |  |  | 0.0001 | < 0.0001 |
|  |  |  |  |  |  |  |  |  |

^1^ The multivariable Cox regression model initially included sex, age, year of diagnosis, family history of colorectal cancer, tumour location, disease stage, tumour differentiation, microsatellite instability, CpG island methylator phenotype, *KRAS* mutation*, BRAF* mutation, *PIK3CA* mutation, long-interspersed nucleotide element-1 methylation level, PTGS2 (cyclooxygenase-2) expression, and nuclear CTNNB1 (beta-catenin) expression. A backward elimination with a threshold *P* of 0.05 was used to select variables for the final models.

^2^ *P*_trend_ value was calculated across the ordinal categories (low, intermediate, and high) of overall lymphocytic reaction score in the Cox regression model.

Abbreviations: CI, confidence interval; HR, hazard ratio.

Supplementary Table S6. Overall lymphocytic reaction score and patient survival in colorectal cancer cases with neoantigen load data

|  |  | Colorectal cancer-specific survival | | |  | Overall survival | | |
| --- | --- | --- | --- | --- | --- | --- | --- | --- |
|  | No. of  cases | No. of  events | Univariable  HR (95% CI)^1^ | Multivariable  HR (95% CI)^1,2^ |  | No. of  events | Univariable  HR (95% CI)^1^ | Multivariable  HR (95% CI)^1,2^ |
|  |  |  |  |  |  |  |  |  |
| Overall lymphocytic reaction score |  |  |  |  |  |  |  |  |
| Low (0-2) | 289 | 102 | 1 (referent) | 1 (referent) |  | 183 | 1 (referent) | 1 (referent) |
| Intermediate (3-6) | 174 | 33 | 0.44 (0.29-0.67) | 0.53 (0.34-0.82) |  | 84 | 0.64 (0.48-0.85) | 0.59 (0.44-0.80) |
| High (7-12) | 42 | 4 | 0.27 (0.09-0.81) | 0.31 (0.08-1.18) |  | 24 | 0.53 (0.30-0.91) | 0.50 (0.28-0.92) |
| *P*_trend_^3^ |  |  | < 0.0001 | 0.0048 |  |  | 0.0007 | 0.0016 |
|  |  |  |  |  |  |  |  |  |

^1^ IPW was applied to reduce a bias due to the availability of tumour tissue after cancer diagnosis (see “Statistical analysis” subsection for details).

^2^ The multivariable Cox regression model initially included sex, age, year of diagnosis, family history of colorectal cancer, tumour location, disease stage, tumour differentiation, microsatellite instability, CpG island methylator phenotype, *KRAS* mutation*, BRAF* mutation, *PIK3CA* mutation, long-interspersed nucleotide element-1 methylation level, PTGS2 (cyclooxygenase-2) expression, nuclear CTNNB1 (beta-catenin) expression, and neoantigen load. A backward elimination with a threshold *P* of 0.05 was used to select variables for the final models.

^3^ *P*_trend_ value was calculated across the ordinal categories (low, intermediate, and high) of overall lymphocytic reaction score in the IPW-adjusted Cox regression model.

Abbreviations: CI, confidence interval; HR, hazard ratio; IPW, inverse probability weighting.

Supplementary Table S7. Lymphocytic reaction components and patient survival in strata of year of diagnosis

|  |  | Colorectal cancer-specific survival | | |  | Overall survival | | |
| --- | --- | --- | --- | --- | --- | --- | --- | --- |
|  | No. of  cases | No. of  events | Univariable  HR (95% CI)^1^ | Multivariable  HR (95% CI)^1,2^ |  | No. of  events | Univariable  HR (95% CI)^1^ | Multivariable  HR (95% CI)^1,2^ |
|  |  |  |  |  |  |  |  |  |
| **Diagnosed in 1995 or before** |  |  |  |  |  |  |  |  |
| Crohn’s-like lymphoid reaction | | |  |  |  |  |  |  |
| Negative/low | 326 | 115 | 1 (referent) | 1 (referent) |  | 233 | 1 (referent) | 1 (referent) |
| Intermediate/high | 89 | 16 | 0.44 (0.24-0.78) | 0.43 (0.23-0.79) |  | 60 | 0.55 (0.35-0.88) | 0.52 (0.32-0.84) |
|  |  |  |  |  |  |  |  |  |
| **Diagnosed in 1996 to 2000** |  |  |  |  |  |  |  |  |
| Crohn’s-like lymphoid reaction | | |  |  |  |  |  |  |
| Negative/low | 233 | 92 | 1 (referent) | 1 (referent) |  | 153 | 1 (referent) | 1 (referent) |
| Intermediate/high | 89 | 19 | 0.48 (0.29-0.81) | 0.53 (0.31-0.91) |  | 53 | 0.59 (0.40-0.87) | 0.61 (0.41-0.92) |
|  |  |  |  |  |  |  |  |  |
| **Diagnosed in 2001 to 2008** |  |  |  |  |  |  |  |  |
| Crohn’s-like lymphoid reaction | | |  |  |  |  |  |  |
| Negative/low | 344 | 98 | 1 (referent) | 1 (referent) |  | 177 | 1 (referent) | 1 (referent) |
| Intermediate/high | 114 | 12 | 0.33 (0.18-0.62) | 0.53 (0.29-0.97) |  | 49 | 0.69 (0.51-0.92) | 0.89 (0.65-1.22) |
|  |  |  |  |  |  |  |  |  |
| *P*_interaction_^3^ |  |  | 0.65 | 0.49 |  |  | 0.35 | 0.023 |
|  |  |  |  |  |  |  |  |  |
|  |  |  |  |  |  |  |  |  |
| **Diagnosed in 1995 or before** |  |  |  |  |  |  |  |  |
| Peritumoural lymphocytic reaction | | |  |  |  |  |  |  |
| Negative/low | 40 | 20 | 1 (referent) | 1 (referent) |  | 29 | 1 (referent) | 1 (referent) |
| Intermediate/high | 480 | 145 | 0.67 (0.40-1.13) | 0.57 (0.35-0.93) |  | 340 | 0.84 (0.49-1.45) | 0.63 (0.37-1.08) |
|  |  |  |  |  |  |  |  |  |
| **Diagnosed in 1996 to 2000** |  |  |  |  |  |  |  |  |
| Peritumoural lymphocytic reaction | | |  |  |  |  |  |  |
| Negative/low | 49 | 38 | 1 (referent) | 1 (referent) |  | 41 | 1 (referent) | 1 (referent) |
| Intermediate/high | 391 | 109 | 0.25 (0.17-0.36) | 0.31 (0.21-0.46) |  | 230 | 0.30 (0.20-0.45) | 0.38 (0.25-0.56) |
|  |  |  |  |  |  |  |  |  |
| **Diagnosed in 2001 to 2008** |  |  |  |  |  |  |  |  |
| Peritumoural lymphocytic reaction | | |  |  |  |  |  |  |
| Negative/low | 121 | 49 | 1 (referent) | 1 (referent) |  | 76 | 1 (referent) | 1 (referent) |
| Intermediate/high | 375 | 68 | 0.43 (0.30-0.63) | 0.62 (0.41-0.92) |  | 165 | 0.63 (0.48-0.83) | 0.77 (0.57-1.03) |
|  |  |  |  |  |  |  |  |  |
| *P*_interaction_^3^ |  |  | 0.82 | 0.11 |  |  | 0.68 | 0.018 |
|  |  |  |  |  |  |  |  |  |
|  |  |  |  |  |  |  |  |  |
| **Diagnosed in 1995 or before** |  |  |  |  |  |  |  |  |
| Intratumoural perigrandular reaction | | |  |  |  |  |  |  |
| Negative/low | 32 | 16 | 1 (referent) | 1 (referent) |  | 23 | 1 (referent) | 1 (referent) |
| Intermediate/high | 488 | 149 | 0.62 (0.36-1.07) | 0.58 (0.34-1.00) |  | 346 | 0.76 (0.42-1.38) | 0.64 (0.35-1.17) |
|  |  |  |  |  |  |  |  |  |
| **Diagnosed in 1996 to 2000** |  |  |  |  |  |  |  |  |
| Intratumoural perigrandular reaction | | |  |  |  |  |  |  |
| Negative/low | 44 | 31 | 1 (referent) | 1 (referent) |  | 35 | 1 (referent) | 1 (referent) |
| Intermediate/high | 396 | 116 | 0.29 (0.19-0.43) | 0.30 (0.21-0.42) |  | 236 | 0.35 (0.23-0.51) | 0.35 (0.25-0.49) |
|  |  |  |  |  |  |  |  |  |
| **Diagnosed in 2001 to 2008** |  |  |  |  |  |  |  |  |
| Intratumoural perigrandular reaction | |  |  |  |  |  |  |  |
| Negative/low | 117 | 44 | 1 (referent) | 1 (referent) |  | 66 | 1 (referent) | 1 (referent) |
| Intermediate/high | 385 | 74 | 0.45 (0.31-0.66) | 0.63 (0.42-0.93) |  | 177 | 0.72 (0.54-0.96) | 0.87 (0.64-1.18) |
|  |  |  |  |  |  |  |  |  |
| *P*_interaction_^3^ |  |  | 0.68 | 0.25 |  |  | 0.66 | 0.043 |
|  |  |  |  |  |  |  |  |  |
|  |  |  |  |  |  |  |  |  |
| **Diagnosed in 1995 or before** |  |  |  |  |  |  |  |  |
| Tumour-infiltrating lymphocytes | | |  |  |  |  |  |  |
| Negative/low | 411 | 138 | 1 (referent) | 1 (referent) |  | 293 | 1 (referent) | 1 (referent) |
| Intermediate/high | 109 | 27 | 0.77 (0.49-1.21) | 0.67 (0.42-1.08) |  | 76 | 0.99 (0.68-1.44) | 0.89 (0.60-1.33) |
|  |  |  |  |  |  |  |  |  |
| **Diagnosed in 1996 to 2000** |  |  |  |  |  |  |  |  |
| Tumour-infiltrating lymphocytes | | |  |  |  |  |  |  |
| Negative/low | 324 | 120 | 1 (referent) | 1 (referent) |  | 200 | 1 (referent) | 1 (referent) |
| Intermediate/high | 115 | 27 | 0.51 (0.33-0.79) | 0.44 (0.27-0.71) |  | 71 | 0.58 (0.41-0.82) | 0.48 (0.33-0.71) |
|  |  |  |  |  |  |  |  |  |
| **Diagnosed in 2001 to 2008** |  |  |  |  |  |  |  |  |
| Tumour-infiltrating lymphocytes |  |  |  |  |  |  |  |  |
| Negative/low | 360 | 98 | 1 (referent) | 1 (referent) |  | 177 | 1 (referent) | 1 (referent) |
| Intermediate/high | 142 | 20 | 0.47 (0.28-0.77) | 0.61 (0.36-1.03) |  | 66 | 0.81 (0.61-1.06) | 0.91 (0.66-1.24) |
|  |  |  |  |  |  |  |  |  |
| *P*_interaction_^3^ |  |  | 0.030 | 0.28 |  |  | 0.39 | 0.99 |
|  |  |  |  |  |  |  |  |  |

^1^ IPW was applied to reduce a bias due to the availability of tumour tissue after cancer diagnosis (see “Statistical analysis” subsection for details).

^2^ The multivariable Cox regression model initially included sex, age, family history of colorectal cancer, tumour location, disease stage, tumour differentiation, microsatellite instability, CpG island methylator phenotype, *KRAS* mutation*, BRAF* mutation, *PIK3CA* mutation, long-interspersed nucleotide element-1 methylation level, PTGS2 (cyclooxygenase-2) expression, and nuclear CTNNB1 (beta-catenin) expression. A backward elimination with a threshold *P* of 0.05 was used to select variables for the final models.

^3^ *P*_interaction_ value (two-sided) was calculated using the Wald test for the cross-product of the ordinal category (negative/low, intermediate, and high) of each lymphocytic reaction component and year of diagnosis (1995 or before, 1996-2000, and 2001-2008) in the IPW-adjusted Cox regression model.

Abbreviations: CI, confidence interval; HR, hazard ratio; IPW, inverse probability weighting.

Supplementary Table S8. Lymphocytic reaction components and patient survival in strata of tumour location

|  |  | Colorectal cancer-specific survival | | |  | Overall survival | | |
| --- | --- | --- | --- | --- | --- | --- | --- | --- |
|  | No. of  cases | No. of  events | Univariable  HR (95% CI)^1^ | Multivariable  HR (95% CI)^1,2^ |  | No. of  events | Univariable  HR (95% CI)^1^ | Multivariable  HR (95% CI)^1,2^ |
|  |  |  |  |  |  |  |  |  |
| **Proximal colon** |  |  |  |  |  |  |  |  |
| Crohn’s-like lymphoid reaction | | |  |  |  |  |  |  |
| Negative/low | 393 | 128 | 1 (referent) | 1 (referent) |  | 247 | 1 (referent) | 1 (referent) |
| Intermediate/high | 195 | 30 | 0.41 (0.27-0.64) | 0.51 (0.32-0.81) |  | 111 | 0.64 (0.49-0.85) | 0.75 (0.56-1.02) |
|  |  |  |  |  |  |  |  |  |
| **Distal colon** |  |  |  |  |  |  |  |  |
| Crohn’s-like lymphoid reaction | | |  |  |  |  |  |  |
| Negative/low | 286 | 94 | 1 (referent) | 1 (referent) |  | 168 | 1 (referent) | 1 (referent) |
| Intermediate/high | 67 | 8 | 0.31 (0.14-0.65) | 0.28 (0.12-0.64) |  | 39 | 0.49 (0.31-0.79) | 0.43 (0.27-0.69) |
|  |  |  |  |  |  |  |  |  |
| **Rectum** |  |  |  |  |  |  |  |  |
| Crohn’s-like lymphoid reaction | | |  |  |  |  |  |  |
| Negative/low | 221 | 82 | 1 (referent) | 1 (referent) |  | 147 | 1 (referent) | 1 (referent) |
| Intermediate/high | 29 | 8 | 0.57 (0.28-1.15) | 0.74 (0.37-1.46) |  | 11 | 0.51 (0.26-0.99) | 0.63 (0.32-1.25) |
|  |  |  |  |  |  |  |  |  |
| *P*_interaction_^3^ |  |  | 0.79 | 0.92 |  |  | 0.31 | 0.27 |
|  |  |  |  |  |  |  |  |  |
|  |  |  |  |  |  |  |  |  |
| **Proximal colon** |  |  |  |  |  |  |  |  |
| Peritumoural lymphocytic reaction | | |  |  |  |  |  |  |
| Negative/low | 93 | 49 | 1 (referent) | 1 (referent) |  | 66 | 1 (referent) | 1 (referent) |
| Intermediate/high | 609 | 152 | 0.43 (0.30-0.62) | 0.48 (0.34-0.68) |  | 370 | 0.58 (0.42-0.81) | 0.68 (0.49-0.94) |
|  |  |  |  |  |  |  |  |  |
| **Distal colon** |  |  |  |  |  |  |  |  |
| Peritumoural lymphocytic reaction | | |  |  |  |  |  |  |
| Negative/low | 67 | 33 | 1 (referent) | 1 (referent) |  | 46 | 1 (referent) | 1 (referent) |
| Intermediate/high | 369 | 90 | 0.51 (0.33-0.79) | 0.53 (0.33-0.84) |  | 208 | 0.54 (0.37-0.80) | 0.58 (0.39-0.85) |
|  |  |  |  |  |  |  |  |  |
| **Rectum** |  |  |  |  |  |  |  |  |
| Peritumoural lymphocytic reaction | | |  |  |  |  |  |  |
| Negative/low | 47 | 23 | 1 (referent) | 1 (referent) |  | 32 | 1 (referent) | 1 (referent) |
| Intermediate/high | 265 | 78 | 0.49 (0.30-0.79) | 0.37 (0.23-0.61) |  | 155 | 0.51 (0.33-0.79) | 0.42 (0.28-0.63) |
|  |  |  |  |  |  |  |  |  |
| *P*_interaction_^3^ |  |  | 0.27 | 0.83 |  |  | 0.66 | 0.21 |
|  |  |  |  |  |  |  |  |  |
|  |  |  |  |  |  |  |  |  |
| **Proximal colon** |  |  |  |  |  |  |  |  |
| Intratumoural perigrandular reaction | | |  |  |  |  |  |  |
| Negative/low | 88 | 40 | 1 (referent) | 1 (referent) |  | 58 | 1 (referent) | 1 (referent) |
| Intermediate/high | 615 | 161 | 0.51 (0.35-0.74) | 0.54 (0.38-0.78) |  | 379 | 0.67 (0.48-0.94) | 0.76 (0.53-1.07) |
|  |  |  |  |  |  |  |  |  |
| **Distal colon** |  |  |  |  |  |  |  |  |
| Intratumoural perigrandular reaction | | |  |  |  |  |  |  |
| Negative/low | 62 | 28 | 1 (referent) | 1 (referent) |  | 37 | 1 (referent) | 1 (referent) |
| Intermediate/high | 377 | 95 | 0.52 (0.33-0.81) | 0.47 (0.29-0.75) |  | 217 | 0.58 (0.39-0.87) | 0.55 (0.37-0.83) |
|  |  |  |  |  |  |  |  |  |
| **Rectum** |  |  |  |  |  |  |  |  |
| Intratumoural perigrandular reaction | |  |  |  |  |  |  |  |
| Negative/low | 41 | 22 | 1 (referent) | 1 (referent) |  | 28 | 1 (referent) | 1 (referent) |
| Intermediate/high | 273 | 80 | 0.41 (0.26-0.64) | 0.41 (0.26-0.66) |  | 160 | 0.45 (0.29-0.69) | 0.49 (0.32-0.74) |
|  |  |  |  |  |  |  |  |  |
| *P*_interaction_^3^ |  |  | 0.85 | 0.70 |  |  | 0.21 | 0.16 |
|  |  |  |  |  |  |  |  |  |
|  |  |  |  |  |  |  |  |  |
| **Proximal colon** |  |  |  |  |  |  |  |  |
| Tumour-infiltrating lymphocytes | | |  |  |  |  |  |  |
| Negative/low | 451 | 152 | 1 (referent) | 1 (referent) |  | 284 | 1 (referent) | 1 (referent) |
| Intermediate/high | 251 | 48 | 0.53 (0.37-0.76) | 0.58 (0.39-0.84) |  | 152 | 0.79 (0.62-1.01) | 0.84 (0.63-1.11) |
|  |  |  |  |  |  |  |  |  |
| **Distal colon** |  |  |  |  |  |  |  |  |
| Tumour-infiltrating lymphocytes | | |  |  |  |  |  |  |
| Negative/low | 366 | 109 | 1 (referent) | 1 (referent) |  | 213 | 1 (referent) | 1 (referent) |
| Intermediate/high | 74 | 14 | 0.57 (0.32-1.02) | 0.54 (0.28-1.03) |  | 41 | 0.72 (0.47-1.11) | 0.62 (0.39-1.00) |
|  |  |  |  |  |  |  |  |  |
| **Rectum** |  |  |  |  |  |  |  |  |
| Tumour-infiltrating lymphocytes |  |  |  |  |  |  |  |  |
| Negative/low | 273 | 92 | 1 (referent) | 1 (referent) |  | 170 | 1 (referent) | 1 (referent) |
| Intermediate/high | 40 | 11 | 0.59 (0.31-1.13) | 0.57 (0.28-1.14) |  | 19 | 0.63 (0.36-1.10) | 0.57 (0.32-1.02) |
|  |  |  |  |  |  |  |  |  |
| *P*_interaction_^3^ |  |  | 0.93 | 0.73 |  |  | 0.15 | 0.079 |
|  |  |  |  |  |  |  |  |  |

^1^ IPW was applied to reduce a bias due to the availability of tumour tissue after cancer diagnosis (see “Statistical analysis” subsection for details).

^2^ The multivariable Cox regression model initially included sex, age, year of diagnosis, family history of colorectal cancer, disease stage, tumour differentiation, microsatellite instability, CpG island methylator phenotype, *KRAS* mutation*, BRAF* mutation, *PIK3CA* mutation, long-interspersed nucleotide element-1 methylation level, PTGS2 (cyclooxygenase-2) expression, and nuclear CTNNB1 (beta-catenin) expression. A backward elimination with a threshold *P* of 0.05 was used to select variables for the final models.

^3^ *P*_interaction_ value (two-sided) was calculated using the Wald test for the cross-product of the ordinal category (negative/low, intermediate, and high) of each lymphocytic reaction component and tumour location (proximal colon, distal colon, and rectum) in the IPW-adjusted Cox regression model.

Abbreviations: CI, confidence interval; HR, hazard ratio; IPW, inverse probability weighting.

Supplementary Table S9. The prognostic interactions between the lymphocytic reaction components in relation to colorectal cancer-specific survival in the multivariable model

| Lymphocytic reaction Components^1,2,3^ | Crohn’s-like lymphoid reaction | Peritumoural lymphocytic reaction | Intratumoural perigrandular reaction | Tumour-infiltrating lymphocytes |
| --- | --- | --- | --- | --- |
| Crohn’s-like lymphoid reaction |  | *P*_interaction_=0.30 | *P*_interaction_=0.47 | *P*_interaction_=0.15 |
| Peritumoural lymphocytic reaction | *P*_interaction_=0.30 |  | *P*_interaction_=0.88 | *P*_interaction_=0.99 |
| Intratumoural perigrandular reaction | *P*_interaction_=0.47 | *P*_interaction_=0.88 |  | *P*_interaction_=0.39 |
| Tumour-infiltrating lymphocytes | *P*_interaction_=0.15 | *P*_interaction_=0.99 | *P*_interaction_=0.39 |  |

^1^ IPW was applied to reduce a bias due to the availability of tumour tissue after cancer diagnosis (see “Statistical analysis” subsection for details).

^2^ The multivariable Cox regression model initially included sex, age, year of diagnosis, family history of colorectal cancer, tumour location, disease stage, tumour differentiation, CpG island methylator phenotype, *KRAS* mutation*, BRAF* mutation, *PIK3CA* mutation, long-interspersed nucleotide element-1 methylation level, PTGS2 (cyclooxygenase-2) expression, and nuclear CTNNB1 (beta-catenin) expression. A backward elimination with a threshold *P* of 0.05 was used to select variables for the final models.

^3^ *P*_interaction_ value (two-sided) was calculated using the Wald test for the cross-product of the ordinal categories (negative/low, intermediate, and high) of two lymphocytic reaction components in the IPW-adjusted Cox regression model.

Abbreviations: IPW, inverse probability weighting.

Supplementary Table S10. The prognostic interactions between tumour-infiltrating lymphocytes and Crohn’s-like lymphoid reaction

|  |  | Colorectal cancer-specific survival | | |  | Overall survival | | |
| --- | --- | --- | --- | --- | --- | --- | --- | --- |
|  | No. of  cases | No. of  events | Univariable  HR (95% CI)^1^ | Multivariable  HR (95% CI)^1,2^ |  | No. of  events | Univariable  HR (95% CI)^1^ | Multivariable  HR (95% CI)^1,2^ |
|  |  |  |  |  |  |  |  |  |
| **Tumour-infiltrating lymphocytes-negative/low** | | | | |  |  |  |  |
| Crohn’s-like lymphoid reaction | | |  |  |  |  |  |  |
| Negative/low | 784 | 273 | 1 (referent) | 1 (referent) |  | 494 | 1 (referent) | 1 (referent) |
| Intermediate/high | 110 | 18 | 0.41 (0.25-0.68) | 0.43 (0.25-0.74) |  | 53 | 0.51 (0.35-0.74) | 0.53 (0.36-0.78) |
|  |  |  |  |  |  |  |  |  |
| **Tumour-infiltrating lymphocytes-intermediate/high** | | | | |  |  |  |  |
| Crohn’s-like lymphoid reaction | | |  |  |  |  |  |  |
| Negative/low | 117 | 30 | 1 (referent) | 1 (referent) |  | 67 | 1 (referent) | 1 (referent) |
| Intermediate/high | 182 | 29 | 0.59 (0.34-1.03) | 0.69 (0.38-1.25) |  | 109 | 0.77 (0.53-1.10) | 0.88 (0.59-1.33) |
|  |  |  |  |  |  |  |  |  |
| *P*_interaction_^3^ |  |  | 0.13 | 0.15 |  |  | 0.10 | 0.13 |
|  |  |  |  |  |  |  |  |  |
|  |  |  |  |  |  |  |  |  |
| **Crohn’s-like lymphoid reaction-negative/low** | | | | |  |  |  |  |
| Tumour-infiltrating lymphocytes | | |  |  |  |  |  |  |
| Negative/low | 784 | 273 | 1 (referent) | 1 (referent) |  | 494 | 1 (referent) | 1 (referent) |
| Intermediate/high | 117 | 30 | 0.68 (0.46-1.01) | 0.69 (0.44-1.07) |  | 67 | 0.90 (0.67-1.21) | 0.78 (0.55-1.11) |
|  |  |  |  |  |  |  |  |  |
| **Crohn’s-like lymphoid reaction-intermediate/high** | | | | |  |  |  |  |
| Tumour-infiltrating lymphocytes | | |  |  |  |  |  |  |
| Negative/low | 110 | 18 | 1 (referent) | 1 (referent) |  | 53 | 1 (referent) | 1 (referent) |
| Intermediate/high | 182 | 29 | 0.98 (0.52-1.85) | 1.09 (0.55-2.16) |  | 109 | 1.35 (0.88-2.06) | 1.30 (0.83-2.05) |
|  |  |  |  |  |  |  |  |  |
| *P*_interaction_^3^ |  |  | 0.13 | 0.15 |  |  | 0.10 | 0.13 |
|  |  |  |  |  |  |  |  |  |

^1^ IPW was applied to reduce a bias due to the availability of tumour tissue after cancer diagnosis (see “Statistical analysis” subsection for details).

^2^ The multivariable Cox regression model initially included sex, age, year of diagnosis, family history of colorectal cancer, tumour location, disease stage, tumour differentiation, CpG island methylator phenotype, *KRAS* mutation*, BRAF* mutation, *PIK3CA* mutation, long-interspersed nucleotide element-1 methylation level, PTGS2 (cyclooxygenase-2) expression, and nuclear CTNNB1 (beta-catenin) expression. A backward elimination with a threshold *P* of 0.05 was used to select variables for the final models.

^3^ *P*_interaction_ value (two-sided) was calculated using the Wald test for the cross-product of the ordinal categories (negative/low, intermediate, and high) of tumour-infiltrating lymphocytes and Crohn’s-like lymphoid reaction in the IPW-adjusted Cox regression model.

Abbreviations: CI, confidence interval; HR, hazard ratio; IPW, inverse probability weighting.

**Supplementary Figure S1.** Inverse probability weighting-adjusted Kaplan-Meier survival analyses of colorectal cancer patients according to overall lymphocytic reaction score. The *P* values were calculated using the weighted log-rank test for trend (two-sided).


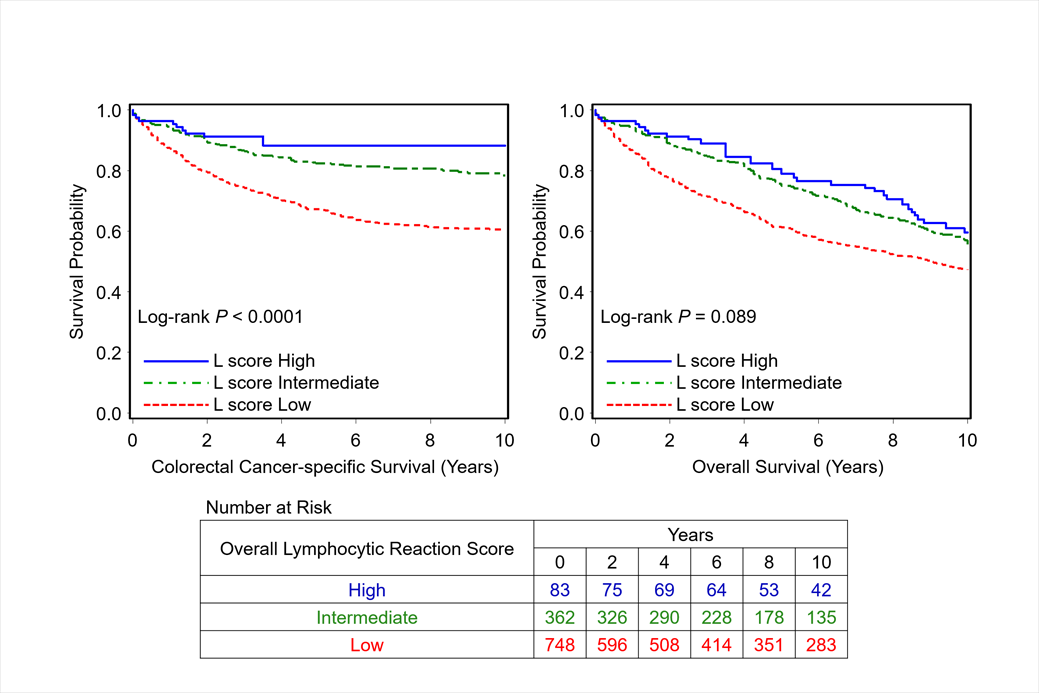

Supplement: Supplementary file 1 — Supplemental tables and figure [file 41416_2020_780_MOESM1_ESM.docx]
